# ROR activation by Nobiletin enhances antitumor efficacy via suppression of IκB/NF-κB signaling in triple-negative breast cancer

**DOI:** 10.1038/s41419-022-04826-5

**Published:** 2022-04-19

**Authors:** Eunju Kim, Yoon-Jin Kim, Zhiwei Ji, Jin Muk Kang, Marvin Wirianto, Keshav Raj Paudel, Joshua A. Smith, Kaori Ono, Jin-Ah Kim, Kristin Eckel-Mahan, Xiaobo Zhou, Hyun Kyoung Lee, Ji Young Yoo, Seung-Hee Yoo, Zheng Chen

**Affiliations:** 1 Department of Biochemistry and Molecular Biology, McGovern Medical School, The University of Texas Health Science Center at Houston (UTHealth), Houston, TX 77030 USA; 2 Center for Computational Systems Medicine, School of Biomedical Informatics, The University of Texas Health Science Center at Houston (UTHealth), Houston, TX 77030 USA; 3 Department of Neurosurgery, McGovern Medical School, University of Texas Health Science Center at Houston (UTHealth), Houston, TX 77030 USA; 4 Department of Pediatrics, Baylor College of Medicine, Houston, TX 77030 USA; 5 Neurological Research Institute, Texas Children’s Hospital, Houston, TX 77030 USA; 6 Lester & Sue Smith Breast Center, Baylor College of Medicine, Houston, TX 77030 USA; 7 Institute of Molecular Medicine, McGovern Medical School, University of Texas Health Science Center at Houston (UTHealth), Houston, TX 77030 USA

**Keywords:** Breast cancer, Circadian rhythms

## Abstract

Triple-negative breast cancer (TNBC) is a heterogeneous disease characterized by poor response to standard therapies and therefore unfavorable clinical outcomes. Better understanding of TNBC and new therapeutic strategies are urgently needed. ROR nuclear receptors are multifunctional transcription factors with important roles in circadian pathways and other processes including immunity and tumorigenesis. Nobiletin (NOB) is a natural compound known to display anticancer effects, and our previous studies showed that NOB activates RORs to enhance circadian rhythms and promote physiological fitness in mice. Here, we identified several TNBC cell lines being sensitive to NOB, by itself or in combination. Cell and xenograft experiments showed that NOB significantly inhibited TNBC cell proliferation and motility in vitro and in vivo. ROR loss- and gain-of-function studies showed concordant effects of the NOB–ROR axis on MDA-MB-231 cell growth. Mechanistically, we found that NOB activates ROR binding to the ROR response elements (RRE) of the IκBα promoter, and NOB strongly inhibited p65 nuclear translocation. Consistent with transcriptomic analysis indicating cancer and NF-κB signaling as major pathways altered by NOB, p65-inducible expression abolished NOB effects, illustrating a requisite role of NF-κB suppression mediating the anti-TNBC effect of NOB. Finally, in vivo mouse xenograft studies showed that NOB enhanced the antitumor efficacy in mammary fat pad implanted TNBC, as a single agent or in combination with the chemotherapy agent Docetaxel. Together, our study highlights an anti-TNBC mechanism of ROR-NOB via suppression of NF-κB signaling, suggesting novel preventive and chemotherapeutic strategies against this devastating disease.

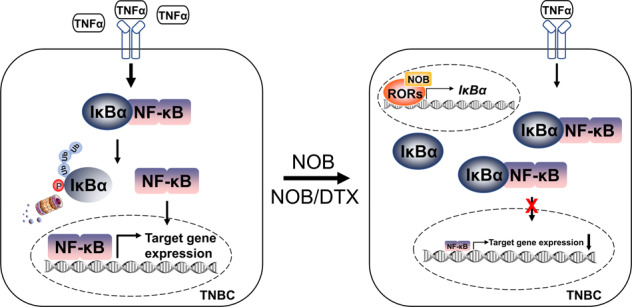

## Introduction

Triple-negative breast cancer (TNBC) is characterized by the lack of expression of estrogen receptor (ER), progesterone receptor (PR), and epidermal growth factor receptor 2 (HER2) [1]. It is a heterogeneous disease that can be subdivided to several subtypes based on histological and molecular features [2]. Compared with other breast cancers, it is resistant to commonly used hormone and targeted therapies, and patients often suffer aggressive tumor progression and less favorable outcomes as a result [1, 3]. Chemotherapies for TNBC, including taxanes and anthracyclines, are typically administered in a sequential single-agent regimen, and combination therapy remains a challenge mainly due to concerns of increased toxicity [1, 4]. Despite being the standard treatment, chemotherapies produce favorable response only in a subset of TNBC patients. Therefore, there is an urgent need for improved therapeutic regimens and mechanistic understanding of TNBC responsiveness to specific agents [3, 5].

Citrus flavonoids, with varying degrees of methoxylation and glycosylation, display diverse beneficial effects in physiology and disease. In polymethoxylated flavonoids (PMFs) including Nobiletin (NOB) and its close analog Tangeretin, the methoxyl groups, in place of hydrogen, confer a more favorable pharmacokinetic profile relative to under-methoxylated counterparts [6–8]. In particular, NOB shows numerous protective effects, including metabolism-promoting, anti-inflammatory and anticancer activities [8–10]. As a chemopreventive agent, NOB exhibits antitumor effects against various cancer cells [11–17]. Interestingly, NOB also functions in a combination setting, acting to sensitize a paclitaxel-resistant A2780 ovarian cancer cell line to the chemotherapeutic agent [16], illustrating a safe and versatile natural compound that warrants further functional and mechanistic investigations.

Our recent studies, along with several others [18–23], reveal a novel mode of action for NOB; specifically, NOB augments the robustness (amplitude) of circadian rhythms, the daily rhythmic processes throughout the body [18]. In metabolic disorders and aging settings where circadian amplitude is attenuated, NOB-treated mice showed marked improvement in metabolism and healthy aging [19, 24], suggesting a key role of NOB as a clock modifier to promote fitness over lifetime. Importantly, filter-binding analysis [18, 19] revealed that RORs (α and γ subtypes) are direct protein targets of NOB. RORs are nuclear receptors functioning in the stabilization loop of the core circadian oscillator [22, 25], providing a mechanistic explanation for the clock-enhancing activity of NOB. Beside circadian rhythms, RORs also regulate a broad array of genes involved in development, metabolism, autoimmunity, cancer, and many other vital processes [26–28]. It remains to be investigated whether and how RORs and the circadian oscillator mediate antitumor efficacies of NOB.

Whereas epidemiological and genetic studies support the notion that circadian disruption increases cancer risk [29, 30], outstanding questions remain regarding mechanistic links and translational implications. The core clock components have shown varying cross-talks with factors involved in tumorigenesis such as cell cycle, DNA damage response, cell migration, and metastasis [25, 29, 31, 32]. Although it has been postulated that the clock largely functions as a tumor suppressor, individual core components show variable effects in cancers, and in some instances appear to promote cancer [33–36]. However, growing evidence strongly suggests anticancer functions of RORs specifically. Consistent with meta-analysis reports linking *RORA* polymorphisms to increased cancer risks [37], our previous TCGA data mining revealed broad downregulation of *ROR* gene expression in multiple cancer types [35]. However, the molecular mechanism for the anticancer function of RORs (particularly RORα and RORγ subtypes) is not fully understood. For example, RORα can be induced by DNA damage and p53 and in turn stabilizes p53, activating its pro-apoptotic function [38]. Furthermore, a synthetic agonist of RORα/γ inhibited breast cancer cell survival and migration, whereas an inverse agonist of RORγ showed opposing effects [39]. Overall, a role of the RORs as a cancer therapeutic target is a promising venue for further studies.

Here, we examined an anticancer role of NOB and the mechanistic function of RORs. We screened pancreatic, lung, and breast cancer cell lines and identified several TNBC cell lines as sensitive to NOB, alone or in combination. Our detailed mechanistic and functional studies illustrate a potent anticancer mechanism of the NOB–ROR axis impinging on nuclear factor-κB (NF-κB), and suggest a promising actionable strategy to improve chemotherapy against TNBC.

## Materials and methods

### Cell culture and transfection

Cells were incubated at 37 °C in a humidified chamber at 5% CO_2_. Lung cancer cells (A549, H441, and H358) were cultured in RPMI-1640 medium (GenDEPOT, TX, USA) and other cells (HPNE-P2M, HPNE-Kras, PNAC1, MCF7, MDA-MB-231, MDA-MB-468, DB7, and BT549) were cultured in DMEM medium (GenDEPOT). All media were supplemented with 10% FBS medium (GenDEPOT) and 1% penicillin–streptomycin medium (GenDEPOT).

Lipofectamine RNAiMAX (Invitrogen, CA, USA) was used for RNA-oligonucleotide transfection, whereas plasmid transfections were performed using Lipofectamine 2000 (Invitrogen) according to the manufacturer’s instructions. All oligonucleotides were purchased from Sigma (MO, USA). The sequence of each gene is shown in follows. *RORA* and *RORC* siRNA are siRORA: 5′-CAAGAUCUGUGGAGACAAAdTdT and siRORC: CGAGGATGAGATTGCCCTCTAdTdT, respectively. For negative control, MISSION® siRNA Universal Negative Controls #2 (Sigma, MO, USA) was used.

*RORA* and *RORC* CRISPR knockdown cells were generated as previous described [40]. Briefly, sgRNAs were designed by using the CPRISRdirect software (https://crispr.dbcls.jp/). MDA-MB-231 cells were transfected with *RORA* and *RORC* sgRNA constructs and selected by puromycin. Colonies were expanded and validated for knockdown efficiency. Experiments were performed by using cells at early passages (before p5).

### Establishment of transfected MDA-MB-231 cells by using Gateway^TM^ cloning

The vector of N-Terminal p3XFLAG-CMV with target cDNA was previously established in our laboratory [18]. We then used a Gateway^TM^ cloning System kit (Invitrogen) for getting the expression vectors. The Gateway donor vector RelA from pDONR221 was recombined into Gateway destination vector pCW 57.1 by LR recombination [41]. The pCW 57.1 (Addgene, #41393) vector alone (Empty vector, Mock) was used as the control. For transient expression, MDA-MB-231 cells were transfected with plasmid constructs (1 μg) by using iMFectin Poly DNA Transfection Reagent (#I7200, GenDEPOT) according to the manufacturer’s instructions.

### Cell proliferation assay

Cell proliferation was measured by WST-1 assays. Briefly, cells were seeded at a density of 1 × 10^4^ cells for WT, *RORA*, or *RORC* CRISPR knockdown, or *RORA*- or *RORC-*overexpressing MDA-MB-231 cells, 5 × 10^3^ cells for p65-overexpressing MDA-MB-231 cells, or 2 × 10^3^ cells for MDA-MB-468 in 96-well plates in complete medium. Twelve hours after seeding, cells were treated with Nobiletin (SelleckChem, TX, USA) or SR1078 (Cayman, UK) according to each study’s protocol. Cell proliferation was detected at 0, 24, 48, and 72 h, respectively. Diluted WST-1(Toronto Research Chemicals Inc., Canada) reagent was added to each well and incubated at 37 °C for 3 h. Absorbance was measured at a wavelength of 450 nm using a Tecan Infinite M200 plate reader (TECAN Life Science, Switzerland).

### Colony formation assay

MDA-MB-231 and MDA-MB-468 cells were seeded at 1000 cells per well onto six-well plates and incubated for 10 days with or without NOB. Culture media were replenished every 3 days. The colonies were fixed using 4% formaldehyde (Sigma) for 10 min, stained with 0.1% (w/v) crystal violet (Sigma) in 10% ethanol for 10 min, and washed with distilled water. Colonies that contained more than 100 cells were counted. Each assay was performed in triplicate.

### Wound-healing assay

For wound-healing assay, cells were seeded into plates in culture medium and cultured until reaching confluent. Twelve hours after pretreatment with NOB, a cell-free area (wound) was constructed using 200 μL pipette tip in each well and washed gently with ice-cold PBS. Then serum-free media containing dimethyl sulfoxide (DMSO) or NOB were added to cells. Healing of the wound was observed after 0, 12, and 16 h by a microscopy (Leica, Wetzlar, Germany) with ×100 magnification and analyzed using ImageJ software (NIH, Bethesda, MD, USA) by the ratio of healing width at each time point to the wound width at 0 h.

### Bioluminescence measurement using TNBC cells

To monitor circadian rhythms in MDA-MB-231 and MDA-MB-468 cells, we transfected and generated clones with stable expression of the Bmal1::Luciferase and Per2::Luciferase reporters under blasticidin selection [42, 43]. U2OS cells were used as a control cell line to rhythmically express these reporters. Cell lines with stable expression were subjected to real-time bioluminescence monitoring by using LumiCycle 32 (Actimetrics, Wilmette, IL, USA). Cells were cultured on 35 mm plates and were synchronized with 200 nM dexamethasone (Sigma) for 1 h. After DMSO or NOB (10 µM) containing recording media [44] were added, the dishes were sealed with vacuum silicon grease and bioluminescence was measured in LumiCycle 32 for continuous bioluminescence monitoring over 4 days. The data were detrended using a first-order polynomial, and then best-fit to a sine wave estimated by a Levenberg–Marquardt algorithm for measurement of circadian parameters in the LumiCycle data analysis program (Actimetrics).

### Flow cytometry analysis

Cells were transfected with si-RORA, si-RORC, and a negative scrambled control (Sigma) and cells were replated 3 × 10^5^ cell/well in six-well plate 12 h after transfection. Cells were treated Nobiletin 10 μM for 12 h after replating. Cells were harvested at 24 h Nobiletin treatment, and fixed in 70% ethanol overnight. DNA staining was performed with a solution of 20 µg/ml propidium iodide containing 10 µg/ml RNase A. Approximately 2 × 10^4^ cells were analyzed by flow cytometry using a Cytomics FC500 flow cytometer running on CXP software (Beckman Coulter, Canada).

### Western blot analysis

Western blotting was performed largely as described previously [45]. Briefly, cells were washed with cold PBS and lysed in HEPES lysis buffer. Protein extracts were separated by SDS polyacrylamide gel electrophoresis and blotted onto a nitrocellulose membrane. Blocking was performed at room temperature for 1 h in TBS-Tween 20 (TBS-T) with 5% blocker (Bio-Rad, CA, USA), followed by incubation with the primary antibodies diluted in TBS-T. After washing with TBS-T, the membrane was incubated with horseradish peroxidase-conjugated secondary antibodies. The protein bands were visualized using a West-Q Pico ECL solution (GenDEPOT). Primary antibodies against the following proteins were used: RORα (ab256799, Abcam); RORγ (sc-293150, Santa Cruz); IκBα, p65 and phospho-p65 ((#9242, #4764, and #3033, Cell Signaling Technology, MA, USA); Flag (A8592), and GAPDH (Sigma). Original blot images are presented in the Supplementary Information.

### Immunocytochemistry

MDA-MB-231 cells were plated and cultured on poly-d-lysine-coated glass coverslips in six-well plates. Twenty-four hours after transfection, the cells were treated with TNF-α (GenScript, NJ, USA) for 15 and 30 min. Then the cells were washed with cold PBS three times and fixed with 4% formaldehyde (Thermo Fisher, MA, USA) in PBS for 15 min at room temperature. They were then rinsed with cold PBS twice and permeabilized with PBS containing 0.1% Triton X-100 (Sigma) for 10 min at room temperature followed by washing with PBS three times. To block the nonspecific binding of the antibodies, samples were incubated with 3% BSA (Invitrogen) in PBS for 1 h at room temperature. Primary antibody against p65 (Cell Signaling Technology) was added to the samples and incubated for overnight at 4 °C. After washing with PBS three times, the cells were incubated with anti-rabbit secondary antibody conjugated with Alexa 488 (Invitrogen) for 1 h at room temperature followed by three times of PBS wash. Cells were stained with a DAPI (1 ng/ml; Sigma) for 5 min. Images were obtained with an LSM700 Confocal microscope (Carl Zeiss, Germany).

### Luciferase reporter assay

MDA-MB-231 cells were seeded in 24-well plates. pGL4.32 [luc2P/NF-κB-RE/Hygro] Vector (Promega, WI, USA) and pSV-β-Galactosidase Control Vector (Promega) were co-transfected using Lipofectamine 2000 (Invitrogen). Cells were treated NOB at 10, 20, and 30 µM for 12 h. β‑Galactosidase was used for normalization. All luciferase measurements were performed with the Luciferase Assay kit (Promega) using a Tecan Infinite M200 plate reader (TECAN Life Science).

### Total RNA extraction and real-time quantitative PCR (qPCR) analysis

Real-time qPCR analysis was conducted as described [46] with minor modifications. Briefly, total RNA was isolated using the PureXtract RNAsol (GenDEPOT). Quantitative reverse transcriptase-PCR was used for determining the mRNA levels of each gene. First, cDNA was synthesized using the amfiRivert cDNA Synthesis Platinum Master Mix (GenDEPOT). The reverse transcription reaction mixture was incubated with the amfiSure SYBR Green qPCR Master Mix (GenDEPOT), followed by real-time amplification and quantitation in Mx3000P qPCR System (Agilent Genomics, CA, USA) according to the manufacturer’s protocol. The fluorescence threshold value was calculated using the MxPro (Agilent Genomics). Data were processed with the comparative cycle threshold method and expressed as fold increase relative to the basal transcription level. Expression of target genes was calculated with the 2^−ΔΔ^Ct method, using GAPDH as a reference. Primers for qPCR analysis are shown in Supplementary Table 1.

### Chromatin immunoprecipitation (ChIP) assays

Cells were fixed in 1% formaldehyde for 10 min at room temperature and neutralized with 125 mmol/L glycine for 5 min. After washing with PBS, cell lysates were sonicated to produce chromatin fragments in 200–500 bp in size. Fragmented chromatins were added into the ChIP dilution buffer (16.7 mM Tris–HCl (pH 8.1), 167 mM NaCl, 1.2 mM EDTA, 1.1% Triton X-100, 0.01% SDS, and inhibitor cocktail). Samples were incubated with anti-RORγ antibody (Santa Cruz, USA) at 4 °C. Immune complexes were precipitated with Protein A resin (Millipore Upstate, MA, USA) and were transferred to mini columns (Bio-Rad) for washing. Finally, the beads were eluted using TE buffer. DNA–protein cross-links were reversed by incubation with 10% Chelex at 95 °C for 10 min. The DNA was treated with proteinase K (Roche, Switzerland) at 55 °C for 30 min. Precipitated chromatins were used as the template for PCR. PCR was carried out using following primer pairs:

RORE1 (Forward 5′-TGGTGGTTGTGGATACCTTGC-3′ and Reverse 5′-ACGATCCTTTTTCTGCGGGA-3′)

RORE2 (Forward 5′-GGCACCCAAATTCGAGGAGA-3′ and Reverse 5′-GGCAGGATGGGACTACCTTG-3′)

RORE3 (Forward 5′-ACTTGGTAGAATTGGTACAGGC-3′ and Reverse 5′-ACAAGGCCAGTCAAGGTAAAGA-3′)

### RNA-seq data analysis

For RNA-seq data analysis, MDA-MB-231 cells was treated 10 µM for 24 h. Total RNA was isolated using the PureXtract RNAsol (GenDEPOT). Two micrograms of extracted RNA samples were used for library construction and RNA-seq analysis (Novogene). The differentially expressed genes (DEGs), with thresholds of *p* < 0.05 and fold change >2, were screened by R package Limma 3.46 [47].

Gene ontology analysis of DEGs was implemented on the integrated platform DAVID [48]. Functional profiles of DEGs were analyzed and visualized by using ClusterProfiler 3.18.1 [49].

### Xenograft studies

All mouse housing and experiments were performed in accordance with the Animal Welfare Committee at University of Texas Health Science Center. Four-week-old outbred female athymic nu/nu mice were obtained from the Jackson Laboratory (Bar Harbor, ME, USA). The mice were randomly divided into four groups and were injected subcutaneously with MDA-MB-231 cells (1 × 10^6^). The five-week-old female FVB mice (Jackson Laboratory) were randomly divided into two groups and injected subcutaneously with DB7 cells (2 × 10^6^). Each cell line was suspended in 50% DPBS/50% growth factor reduced matrigel (Corning, NY, USA) and injected into the abdominal mammary fat pad of nude mice or FVB mice.

After tumor cells were injected, mice were fed with standard chow alone or standard chow supplemented with 0.1% NOB (10 mg/kg b.w.) for the duration of studies. For the combination treatment with DTX, when tumors reached the average size of 150–200 mm^3^, mice were randomized and control DMSO or DTX (10 mg/kg) was administrated by intraperitoneal inject once a week for 4 weeks. Tumor volume (mm^3^) was measured for 43 days after injection of MDA-MB-231 or DB7 cells and calculated using the equation: tumor volume = length × (width)^2^ × 0.5. At the end of the experiments, mice were sacrificed and tumor and plasma were dissected immediately. Interactions between drugs were presented as the combination index (CI), calculated by dividing the expected growth inhibition rate by the observed growth inhibition rate: CI < 1.0 indicates antagonistic cytotoxicity; CI = 1.0 is additive cytotoxicity; and CI > 1.0 is synergistic cytotoxicity.

### TNF-α enzyme-linked immunosorbent assay (ELISA)

For ELISA assay, tumors were dissected immediately from euthanized mice after finishing experiment and stored at −80 °C. Frozen tumor samples were pulverized to get powder tumor. Powder tumor tissues were homogenized in lysis buffer in the presence of a cocktail of proteinase inhibitors (Sigma, MO, USA). Plasma was isolated from whole blood after centrifuge (3000 rpm for 15 min at 4 °C). TNF-α levels of tumor and plasma were detected using TNF-α ELISA kit (R&D Systems, MN, USA) according to the instructions from the manufacturer.

### Immunofluorescence (IF)

Formalin-fixed, paraffin-embedded tumor tissue sections were stained with various antibody for IF. Briefly, the paraffin was removed by Histo-Clear (National Diagnostics, GA, USA). For hydration, the slides were dipped in a gradient of ethanol solution (from 100 to 30%) and in water for 15 min at room temperature. Next, the slides were placed in 0.01 M citric acid buffer (pH 6.0) at 95 °C for 30 min for antigen retrieval. The slides were blocked for 1 h with 5% of goat serum in order to prevent the nonspecific binding of the antibodies and then treated with different specific primary antibodies: Ki67 (Abcam, Cambridge, UK), p-p65 (Cell Signaling, MA, USA), and F4/80 (eBioscience, CA, USA) for overnight at 4 °C followed by incubation with the secondary antibodies (Alexa Fluor 488 goat anti-rabbit IgG (H + L) and goat anti-rat IgG2a-FITC (Invitrogen, CA, USA) for 1 h at room temperature). Nuclei were counter-stained by DAPI Flour mount-G (SouthernBiotech, AL, USA). Images were then captured at least four sections of each samples using a fluorescence microscope (Leica, Germany) and at least four different samples per each group were analyzed by ImageJ program using ImageJ (fluorescent intensity area/total image area × 100) in each experiment.

### Statistical analysis

Data are presented as mean ± SEM unless otherwise indicated. All *n* numbers refer to biologically independent samples. Data were analyzed using Student’s *t*-test, one-way, or two-way ANOVA with Tukey, Dunnett, and Sidak tests for multiple-group comparisons. A *p* value < 0.05 is considered to indicate statistical significance.

## Results

### NOB suppresses TNBC cell growth and motility

**Fig. 1:**
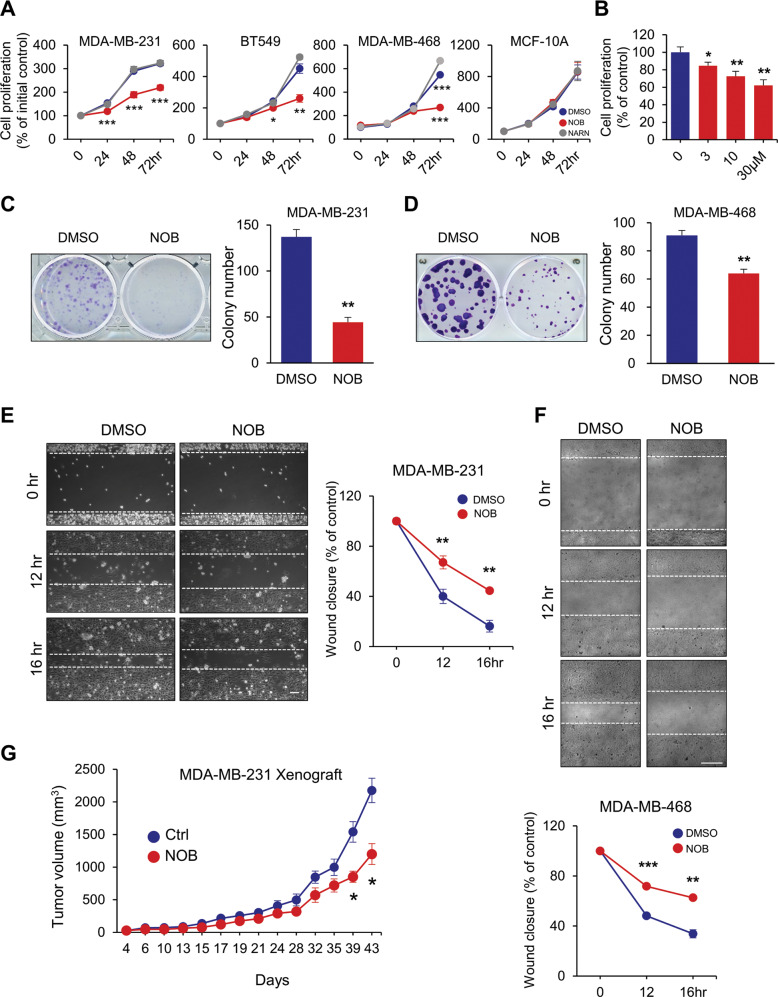
NOB suppress cell survival and motility of TNBC. **A** TNBC cell lines (MDA-MB-231, BT549, and MDA-MB-468) and MCF10A were treated for 24, 48, and 72 h with 10 µM NOB or 0.1% DMSO as control. Cell proliferation was determined by WST-1 assays. Data represent mean ± SEM. Two-tailed Student’s *t*-test shows significant statistical difference between DMSO and NOB. *p < 0.05, **p < 0.01, and ***p < 0.001. **B** MBA-MB-231 cells were treated with various concentrations of NOB (0–30 µM) as indicated for 48 h and subjected to WST-1 assays. Data represent mean ± SEM. Two-tailed Student’s *t*-test, *p < 0.05 and **p < 0.01 vs control. **C** NOB suppressed MDA-MB-231 colony formation. Right panel: quantification. Data represent mean ± SEM. Two-tailed Student’s *t*-test, **p < 0.01. **D** NOB suppressed MDA-MB-468 colony formation. Right panel: quantification. Data represent mean ± SEM. Student *t***-**test, **p < 0.01. **E** Wound-healing assay. The results are expressed as the percentage of motility compared with control cells at 0 h (100%). Data are shown as representative images or mean ± SEM from three independent experiments. Two-tailed Student’s *t*-test, **p < 0.01. Scale bar = 100 µm. **F** Wound-healing assays as above. Data represent mean ± SEM. Student's *t*-test, **p < 0.01, and ***p < 0.001. Scale bar = 277.3 μm. **G** Athymic nude mice were implanted with MDA-MB-231 by mammary fat pad injection. Tumor volume was measured regularly after treatment and the data shown are the mean tumor volumes ± SEM (*n* = 5/group). Two-tailed Student’s *t*-test, *p < 0.05; Ctrl vs NOB.

Having identified the RORs as the direct target of NOB and given that ROR levels are broadly reduced in various cancer types [18, 35], we investigated a possible role of NOB–ROR against cancer. Screening of pancreatic, lung, and breast cancer cell lines identified three TNBC cell lines as highly sensitive to NOB (10 µM) (Supplementary Fig. 1A and Fig. 1A). Specifically, NOB (10 µM) significantly impeded the growth of MDA-MB-231, BT549, and MDA-MB-468 (Fig. 1A, 31.9%, 42.2%, and 50.9% reduction compared to DMSO at 72 h, respectively) without affecting the growth of MCF10A (normal breast epithelial cells) or MCF7 (ER/PR-positive breast cancer cells). In accordance, TCGA database search revealed that ROR expression was significantly reduced in TNBC (Supplementary Fig. 1B). Furthermore, NOB displayed progressive inhibition of MDA-MB-231 proliferation in a dose-dependent manner (Fig. 1B). Next, we examined RORα/γ involvement by examining effects of a previously reported RORα/γ agonist, SR1078 [50]. Similar to NOB, SR1078 was able to curtail MDA-MB-231 cell proliferation by 13.3% (*p* < 0.01) (Supplementary Fig. 1C).

Next, we performed clonogenic and wound-healing assays to assess effects of NOB on colony formation and motility of MDA-MB-231 and MDA-MB-468 cells. We observed that colony formation and colony size were markedly reduced by NOB after 10 days of treatment in MDA-MB-231 (Fig. 1C) and MDA-MB-468 (Fig. 1D) cells. As measured by migration distance, pretreatment of MDA-MB-231 and MDA-MB-468 cells with NOB strongly inhibited their motility (Fig. 1E, F).

Next, we performed xenograft experiments using athymic nude mice. Nude mice, implanted with MDA-MB-231 cells at the left inguinal mammary fat pad, were fed with regular diet (Ctrl) or NOB-containing (0.1%) (NOB) diet. The mean tumor volumes in the mice fed with NOB were significantly reduced to 1199.6 mm^3^ by day 43 compared to 2175.7 mm^3^ in the control mice (*p* < 0.05) (Fig. 1G). These results demonstrate that NOB effectively reduces TNBC cell growth both in vitro and in vivo.

### TNBC cells do not exhibit circadian rhythms

Previously, NOB was found to prevent metabolic syndrome in a circadian clock-dependent manner [18]. Given the circadian dysregulation in tumor cells [51, 52], we investigated circadian rhythms in MDA-MB-231 and MDA-MB-468 cells by monitoring real-time bioluminescence from the introduced stable expression of the Bmal1::Luciferase and Per2::Luciferase (Supplementary Fig. 2A and B, respectively). Compared to the U2OS cells as a positive control, we observed no persistent circadian bioluminescence rhythm, regardless of DMSO or NOB treatment. This agrees with a previous study showing severely disruption of circadian oscillation in MDA-MB-231 cells [53]. The lack of sustained circadian rhythms in MDA-MB-231 cells suggest that the inhibitory effects of NOB may be more directly related to RORs (RORα and RORγ) [39, 54–58], rather than the clock machinery.

### NOB inhibits MDA-MB-231 cell growth via an ROR-dependent mechanism

**Fig. 2:**
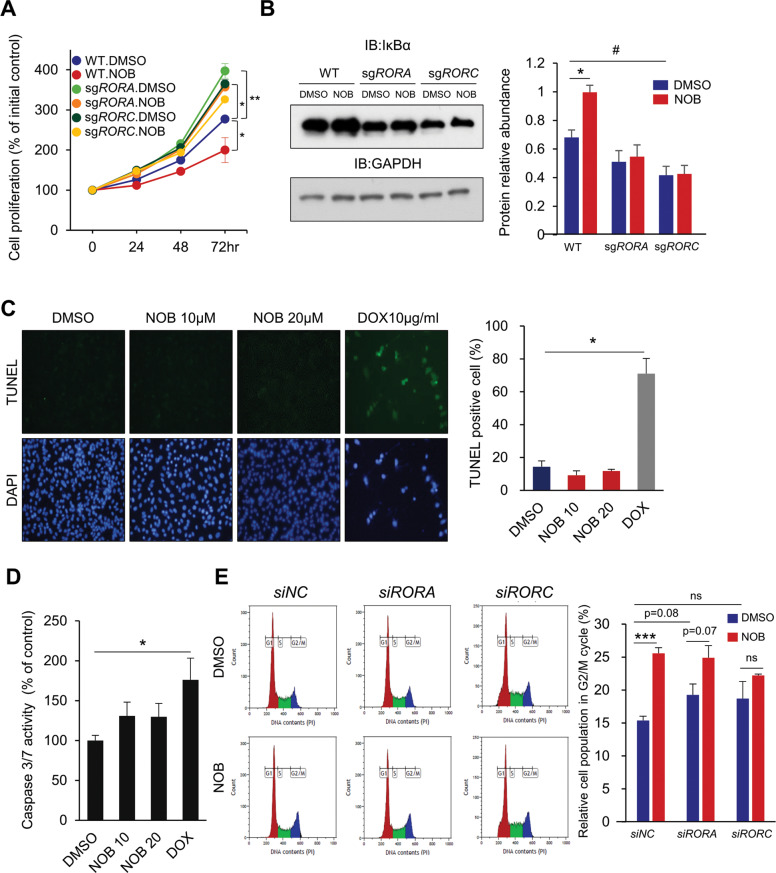
ROR-dependent cell growth is inhibited by NOB. **A** WT and derivative MDA-MB-231 cells with *RORA* or *RORC* knockdown were treated with 10 μM NOB for 24, 48, and 72 h. The data represent mean ± SEM. Two-way ANOVA with Tukey’s multiple comparison, *p < 0.05; **p < 0.01. **B** IκBα protein expression in the above ROR knockdown cells treated with DMSO or 10 µM NOB for 24 h. Right panel: quantification from four experiments. Data represent mean ± SEM. Two-way ANOVA with Sidak’s multiple comparisons test, *p < 0.05, and two-tailed Student’s *t*-test, ^#^*p* < 0.05 WT.DMSO vs sg*RORC*.DMSO. **C** MDA-MB-231 cells were treated with NOB or Doxorubicin (DOX) for 24 h and then stained with TUNEL and DAPI. Two-tailed Student’s *t*-test, *p < 0.05. **D** MDA-MB-231 cells were treated with NOB (10 or 20 µM) or DOX, and apoptosis was examined by detecting caspase-3/7 activity. Data represent the mean ± SEM of three experiments. Two-tailed Student’s *t*-test, *p < 0.05. **E** MDA-MB-231 cells were transfected with control siRNA, siRORA, or siRORC and treated with 10 μM NOB for 24 h. Right panel: quantification data are presented as the percentage of the corresponding phase of the cells and the mean ± SEM. Two-tailed Student’s *t*-test, ***p < 0.001.

Next, we performed loss-of-function experiments by examining cell viability in MDA-MB-231 cells harboring CRISPR-Cas9 sgRNA-mediated *RORA/C* knockdown (Supplementary Fig. 3A). Interestingly, respective ROR knockdown appeared to have reciprocal effects to diminish the level of the other ROR, with a more significant reduction in RORα levels from *RORC* knockdown (Supplementary Fig. 3B). As shown in Fig. 2A, *RORA/C* knockdown increased cell proliferation. NOB showed much diminished cytotoxic effects on *ROR* knockdown cells (*p* < 0.01 for *RORA* knockdown and *p* < 0.05 for *RORC* knockdown cells) in comparison with WT MDA-MB-231 cells where NOB was able to significantly reduce cell proliferation after 48 and 72 h of treatment, demonstrating the ROR dependence of NOB anti-TNBC effects. In accordance, IκBα induction by NOB was abrogated by ROR knockdown (Fig. 2B).

MDA-MB-231 cells, treated with NOB (10 or 20 µM) or Doxorubicin (DOX) 10 µg/ml, were stained with TUNEL and DAPI (Fig. 2C). While DOX-treated cells showed positive TUNEL staining, NOB-treated cells did not. We next evaluated caspase-3/7 activation, and observed significantly increased caspase activation in DOX-treated, but not NOB-treated, cells (Fig. 2D). These results suggest that NOB does not trigger apoptosis in MDA-MB-231 cells. We further examined cell cycle effects of RORs by flow cytometry. NOB treatment significantly increased cell cycle arrest at the G2/M phase (Fig. 2E). In contrast, there is no significant cell cycle effect after siRNA-mediated *ROR* knockdown (Supplementary Fig. 3C), suggesting a role of NOB–ROR in the G2/M checkpoint.

**Fig. 3:**
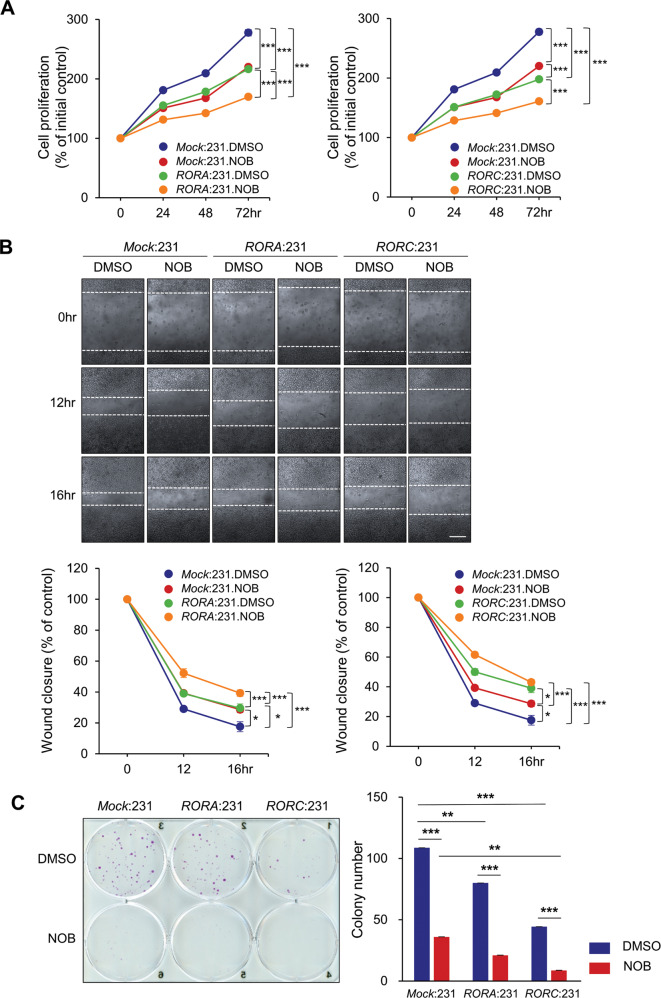
Combination of NOB and ROR ectopic expression in MDA-MB-231 cells suppresses cell growth and motility. **A** WST-1 assays. Mock indicates P3Xflag-CMV10-Neo^r^ (Empty vector). Data represent mean ± SEM. Two-way ANOVA with Sidak’s multiple comparisons test showed significant difference. ***p < 0.001. **B** Wound healing assays showed retarded motility in *RORA*- or *RORC*-expressing MDA-MB-231 cells. The results were expressed as the percentage of the motility of the control cells (100%, upper panel) (×100 magnification, scale bar = 277.3 μm). Data represent mean ± SEM. Two-way ANOVA with Sidak’s multiple comparisons, *p < 0.05 and ***p < 0.001. **C** NOB and *RORA/C* expression in MDA-MB-231 showed a combination effect in colony formation assays. Right panel: quantification. Data represent mean ± SEM. Two-way ANOVA with Sidak’s multiple comparisons test, **p < 0.01 and ***p < 0.001. Interaction between *RORA/C* expression and NOB, *p* = 0.0026 via two-way ANOVA.

We next generated *RORA* or *RORC* overexpressing MDA-MB-231 clones (*RORA*:231 and *RORC*:231). Expression of RORα or RORγ was confirmed by qPCR and western blotting (Supplementary Fig. 3D and E respectively). NOB inhibited cell proliferation dose-dependently in MDA-MB-231 cells transfected with the empty vector (P3Xflag-CMV10-Neo^r^) (Supplementary Fig. 3F). Both *RORA* and *RORC* expression significantly decreased MDA-MB-231 proliferation compared to the control (Fig. 3A, *p* < 0.001 for all at 72 h), and NOB further reduced proliferation of MDA-MB-231 cells expressing *RORA* or *RORC* (*p* < 0.001 for all at 72 h). *RORA* or *RORC* expression also significantly decreased cell motility (Fig. 3B) and colony formation (more significantly by *RORC*, Fig. 3C) compared to control cells. Importantly, NOB displayed strong effects in these ROR-expressing cells, highlighting a prominent role of the NOB–ROR axis against TNBC cells.

### IκBα and NF-κB signaling are direct targets of the NOB–ROR axis

**Fig. 4:**
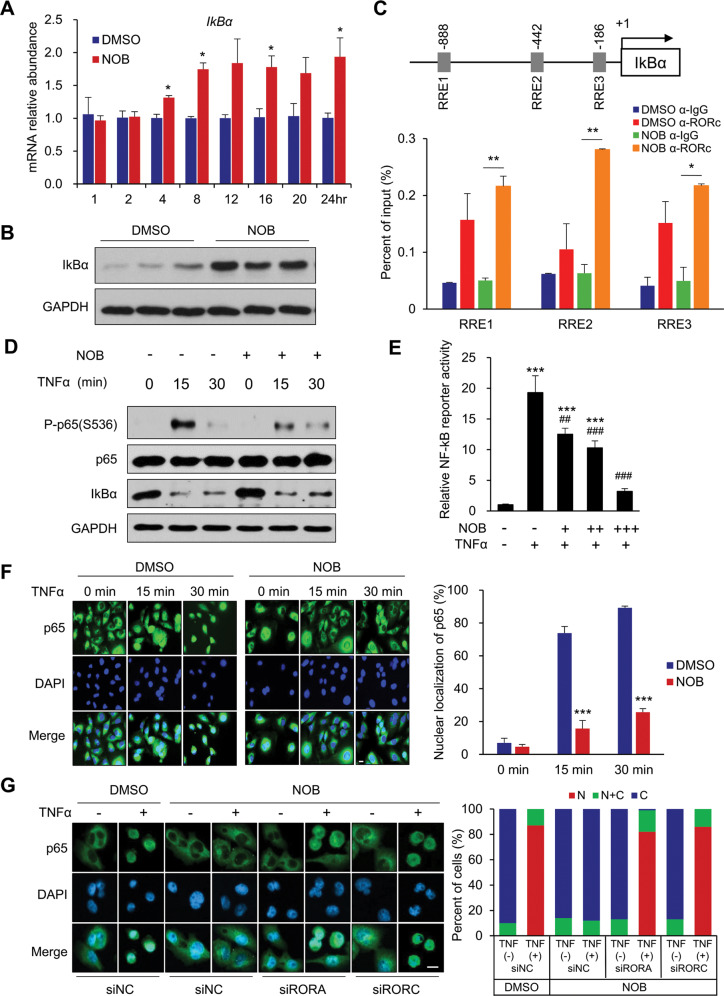
The NF-κB pathway is a direct target of the NOB–RORs axis. **A** MDA-MB-231 cells were treated with NOB. *IkBα* mRNA expression was measured by qRT-PCR. Data represent mean ± SEM. Two-tailed Student’s *t*-test, *p < 0.05. **B** MDA-MB-231 cells were treated with NOB for 24 h. IkBα protein expression was measured by western blotting. In all, 4.4-fold change, *p* < 0.01 compared to DMSO. **C** Schematic representation of putative RRE sites found in the *IκBα* promoter (TRANSFAC). The distance in bp from the transcription start site of *IkBα* gene is shown. MDA-MB-231 cells were treated with 20 µM NOB for 12 h. Samples were normalized to input chromatin and expressed as % input. Error bars represent mean ± SD; *n* = 2; two-tailed Student’s *t*-test. *p < 0.05; **p < 0.01. **D** MDA-MB-231 cells were treated with 10 µM NOB for 12 h prior to TNF-α treatment (10 ng/ml). Whole-cell lysates were subjected to western blot analysis using anti-IκBα, phospho-p65 (Ser536), total p65, and GAPDH antibodies. **E** MDA-MB-231 cells were transfected with pGL4.32 [luc2P/NF-κB-RE/Hygro] vector and treated with NOB (+, 10 µM; ++, 20 µM; +++, 30 µM) for 12 h prior to TNF-α treatment. Data represent mean ± SEM. Two-tailed Student’s *t*-test, ***p < 0.001. Compared with the TNF-α control, ^##^*p* < 0.01, ^###^*p* < 0.001. **F** p65 nuclear localization by TNFα was inhibited by NOB in MDA-MB-231. Representative immunofluorescence images of endogenous p65 in MDA-MB-231 with NOB pretreatment 12 h prior to TNF-α treatment (×400 magnification, scale bar = 10 µm). Two-tailed Student’s *t*-test, ***p < 0.001. **G** MDA-MB-231 cells were transfected with control siRNA, siRORA or siRORC and treated 10 µM NOB for 12 h prior to TNF-α treatment. Representative confocal images are shown (×400 magnification, scale bar = 10 µm). Cell extracts were subjected to dual luciferase assays. N nucleus; C cytoplasm.

Next, we investigated the molecular mechanism underlying NOB effects in MDA-MB-231 cells. Previous studies have identified IκBα, encoded by *NFKBIA*, as a direct transcriptional target of RORα [59]. IκBα is a pivotal regulator of NF-κB signaling [60, 61]. Furthermore, TCGA database analysis showed a significant correlation of *NFKBIA* and *RORC* expression in breast cancer (Supplementary Fig. 4A), suggesting a regulatory role of RORs in *IκBα* transcription regulation. To determine whether *IκBα* transcription is responsive to NOB in MDA-MB-231, we performed qPCR analysis (Fig. 4A) which showed that *IκBα* mRNA expression was activated by NOB. We next examined IκBα protein expression in response to NOB. In MDA-MB-231 cells treated with NOB, IκBα proteins were strongly induced (Fig. 4B).

We determined whether NOB facilitates ROR binding to the consensus ROR response elements (RRE) of the *IκBα* promoter. Based on TRANSFAC analysis (http://genexplain.com/transfac/), we identified three putative RRE sites (Fig. 4C). MDA-MB-231 cells treated 20 µM NOB for 12 h were collected, and chromatin immunoprecipitation (ChIP) was performed by using IgG or anti-RORγ antibody. qPCR amplification revealed that RRE1, RRE2, and RRE3 showed NOB-dependent RORγ recruitment (two-tailed Student’s *t*-test: *p < 0.05; **p < 0.01). These results suggest these RRE elements are functionally involved in ROR binding.

### NOB–ROR represses TNF-α-induced NF-κB activation in MDA-MB-231 cells

To examine the mechanistic role of NOB–ROR in NF-κB signaling in TNBC, we treated MDA-MB-231 cells with NOB (10 µM for 12 h) followed by treatment of tumor necrosis factor α (TNF-α; 10 ng/ml) for 15 and 30 min. Immunoblotting analysis showed that NOB treatment significantly attenuated levels of phosphor-p65 (Ser536) after 15 min of TNF-α treatment (Fig. 4D and Fig. S4B). In accordance, in NF-κB reporter assays using MDA-MB-231 cells transfected with an NRE-luciferase reporter, NOB dose-dependently reduced NF-κB transcriptional activity upon TNF-α induction (10 ng/ml) (Fig. 4E). We next investigated NOB effects on p65 nuclear localization. TNF-α treatment strongly induced endogenous p65 nuclear localization which was inhibited by NOB pretreatment (Fig. 4F).

To investigate the role of RORs in NF-κB signaling, MDA-MB-231 cells were transfected with control siRNA, si-RORA, or si-RORC and treated with NOB (10 μM) for 12 h. While NOB strongly inhibited NF-κB nuclear localization in control siRNA-treated cells, *Ror* knockdown abrogated the NOB effect on p65 nuclear localization in response to TNF-α (Fig. 4G). These results therefore indicate a novel role of ROR-NOB to regulate NF-κB signaling via p65 nuclear localization.

### Inducible p65 expression abolishes NOB inhibition of MDA-MB-231 growth

**Fig. 5:**
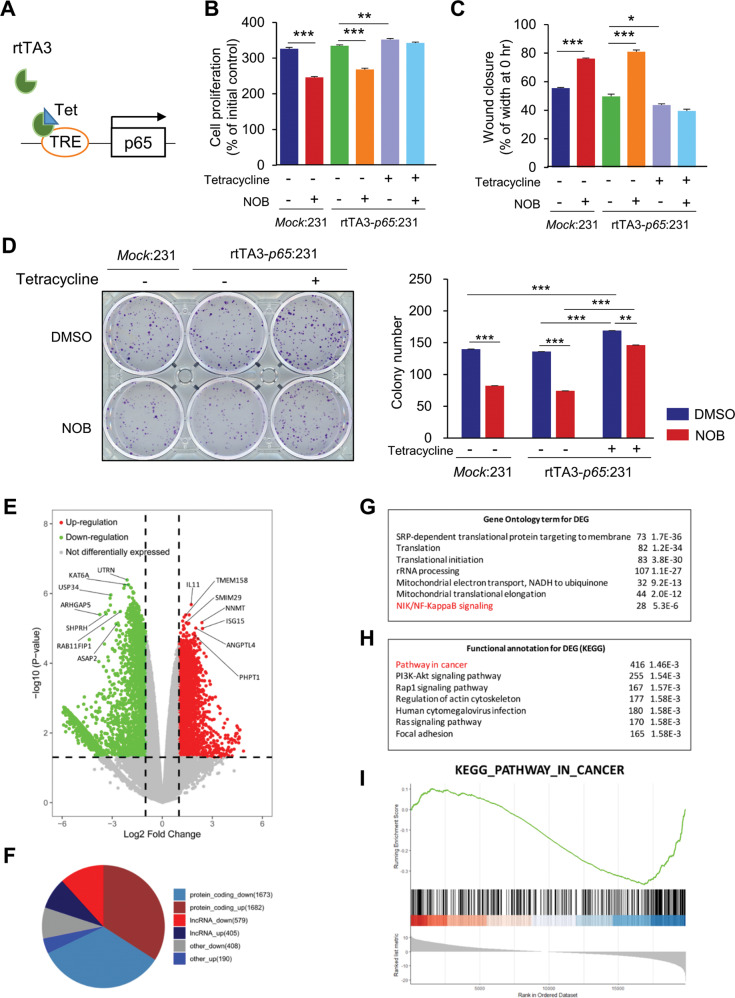
p65 is targeted by NOB to inhibit TNBC. **A** Diagram indicating the p65-inducible system. **B** MDA-MB-231 cells were grown to confluence and treated NOB with (20 µM) and tetracycline (100 nM) for 12 h. WST-1 assays were performed at 72 h after NOB treatment. Mock indicates pCW 57.1 vector (Empty template vector). Data represent mean ± SEM. Two-way ANOVA with Sidak’s multiple comparisons test, **p < 0.01, and ***p < 0.001. **C** Wound-healing assay. The closure of wounds in MDA-MB-231 cells after 16 h of *p65* induction by tetracycline (100 nM). The results are presented as relative motility to the width at 0 h (100%). Data represent mean ± SEM. Two-way ANOVA with Sidak’s multiple comparisons test, *p < 0.05 and ***p < 0.001. **D** Colony formation assays were performed with 20 µM NOB. Data represent mean ± SEM. Two-way ANOVA with Sidak’s multiple comparisons test. **p < 0.01 and ***p < 0.001. **E–I** RNA-seq analysis. **E** Volcano plot of 4937 differential expressed genes (DEGs) between DMSO and NOB treatment MDA-MB-231 cells (*p* < 0.05 and fold change >2). Plots highlighted with red and green represent up- and downregulated genes. Multiple NF-κB pathway genes are marked. **F** Pie chart of protein-coding genes, LncRNA, and other genes in the DEG set. **G** Gene ontology analysis of DEGs with DAVID. Top-ranked pathways are presented, including NF-κB signaling indicated with red. **H** KEGG analysis of DEGs with ClusterProfiler. Top-ranked pathways are presented, including “Pathway in cancer ”. **I** Gene set enrichment analysis demonstrated the enrichment of gene sets related to cancer.

Next, we employed a tet-on system [62, 63] to generate inducible p65 expressing MDA-MB-231 cells (Fig. 5A and Supplementary Fig. 4C). Cell proliferation assays using control and p65-inducible cells showed that p65 induction abolished NOB effects on cell proliferation and motility. Whereas NOB significantly reduced cell proliferation, p65 induction abolished NOB effects on cell proliferation (Fig. 5B) and motility (Fig. 5C and Supplementary Fig. 4D). Consistently, colony formation assay showed p65 induction bypassed NOB effects (Fig. 5D). As expected, IκBα protein induction by NOB was disrupted by p65 overexpression (Supplementary Fig. 4E). qPCR analysis further revealed that expression of multiple p65 target genes in cell proliferation and Wnt/β-catenin signaling was downregulated by NOB, which was abolished in cells overexpressing p65 (Supplementary Fig. 4F). These results suggest that p65 overexpression counteracts NOB to regulate tumor cell proliferation and motility, providing important mechanistic evidence linking NOB–ROR and NF-κB signaling.

### Regulation of cancer and NF-κB pathways in TNBC tumors by NOB–ROR

To delineate changes in the gene expression landscape by NOB in MDA-MB-231 cells, we performed RNA-seq using MDA-MB-231 treated with 10 µM NOB for 24 h and identified large numbers of significantly differentially expressed genes (DEGs; >2-fold change) in response to NOB treatment (Fig. 5E, F). As shown by Volcano plot (Fig. 5E) and pie chart (Fig. 5F), 2660 and 2277 genes were down- and up-regulated by NOB, respectively. Gene ontology analysis showed enrichment of a number of cancer-related cellular pathways, including metabolism/mitochondria, growth and translation, and importantly NF-κB signaling (Fig. 4G–I). By cross-checking the GEO database, we further identified overlapping genes between our differentially expressed genes (DEGs) and reported RORγ target genes in TNBC cells [64] (Supplementary Fig. 5A). These results are consistent with our previous finding linking NOB and mitochondria [19], on the other hand also underscore a pivotal function of NOB–ROR to target the NF-κB pathway in TNBC cells. Of note, IκBα expression was induced by NOB in RNA-seq analysis (fold change = 1.59, *p* = 0.01), consistent with qPCR validation (fold change = 1.62, *p* < 0.05) (Supplementary Fig. 5B).

### Combination of NOB with docetaxel (DTX) or carboplatin (CAR) inhibits TNBC cell growth

**Fig. 6:**
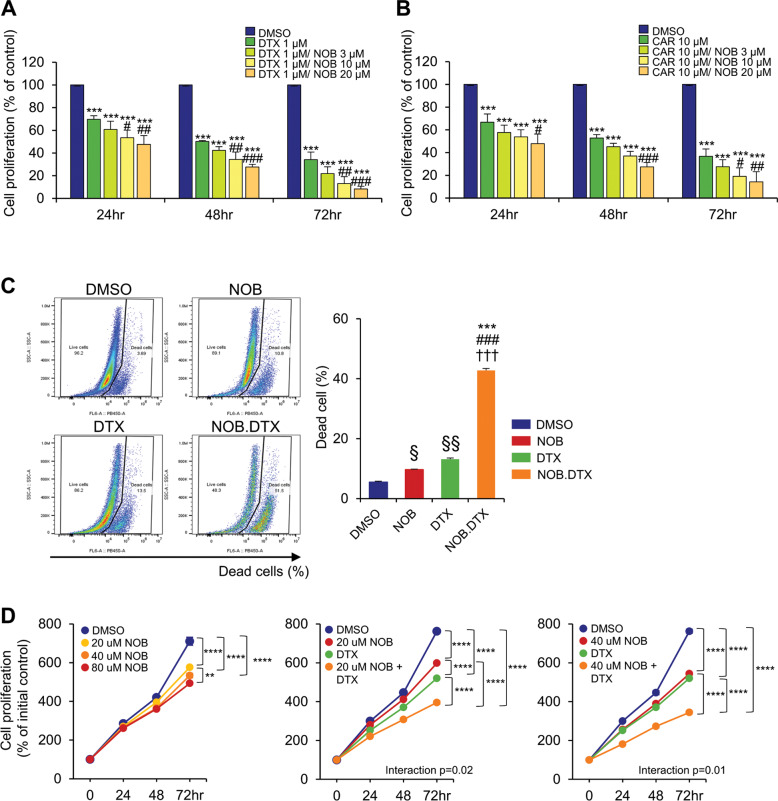
Combination studies show the efficacy of NOB with DTX or CAR in TNBC cells. **A** MDA-MB-231 cells were treated with the NOB and/or DTX for 24, 48, and 72 h and cell proliferation was determined by WST-1 assays. Data represent mean ± SEM. One-way ANOVA with Tukey’s post hoc test showed significant difference compared to DMSO, ***p < 0.001, compared to DTX, ^#^*p* < 0.05, ^##^*p* < 0.01, and ^###^*p* < 0.001. Interaction, *p* < 0.01 via two-way ANOVA. **B** MDA-MB-231 cells were treated with NOB and/or CAR for 24, 48, and 72 h and cell proliferation was determined by WST-1 assay. Data represent mean ± SEM. Two-tailed Student’s *t*-test showed significant difference compared to DMSO, ***p < 0.001, compared to CAR 10 µM, ^#^*p* < 0.05, ^##^*p* < 0.01, and ^###^*p* < 0.001. **C** MDA-MB-468 cells were treated with NOB (100 µM) and/or DTX (5 nM) for 48 h and cell death was determined by FACS analysis. Left panel; Representative images of flow cytometric analysis; right panel; quantification. Data represent mean ± SEM. Two-way ANOVA with Tukey’s multiple comparisons test showed significant difference compared to DMSO, ***p < 0.001, NOB, ^###^*p* < 0.001, and compared to DTX, ^†††^*p* < 0.001 (interaction, *p* < 0.001 via two-way ANOVA). Student *t*-test compared to DMSO, ^§^*p* < 0.05, ^§§^*p* < 0.01. **D** DB7 cells were treated with NOB (20, 40, and 80 µM) and/or DTX (2.5 nM) for 24 and 72 h and cell proliferation was determined by WST-1 assays. Data represent mean ± SEM. Two-way ANOVA with Tukey’s multiple comparisons test showed significant difference compared to DMSO, **p < 0.01; ****p < 0.0001.

To assess the combination efficacy of NOB with a primary chemotherapy, we performed combination treatment of NOB with DTX or CAR and measured MDA-MB-231 cell proliferation. Interestingly, NOB and DTX showed robust combination efficacy (interaction *p* < 0.01 via two-way ANOVA), displaying superior inhibiting effects compared to DTX or CAR alone (Fig. 6A, B). These results suggest that NOB can serve as a strong enhancer for the primary chemotherapy drug.

We further investigated the enhancer effect of NOB in human MDA-MB-468 breast cancer cells and mouse DB7 breast cancer cells. As a control, NOB at 50 and 100 µM doses exhibited no cytotoxicity on MCF10A cells after 48 h of treatment (Supplementary Fig. 6A). In MDA-MB-468 cells, the combination treatment of NOB (100 µM) and DTX (5 nM) for 48 h strongly increased cell death by 69.2% and 77.5% compared to NOB and DTX respectively (Fig. 6C). In comparison, NOB at 50 µM also showed a combination effect with 2.5 nM DTX, albeit to a lesser degree (Supplementary Fig. 6B). In DB7 cells, cell proliferation was attenuated dose-dependently by NOB (Fig. 6D, left), and at the lower doses tested (20 and 40 µM), we detected a significant interaction effect (2-way ANOVA) between NOB and DTX (Fig. 6D). Moreover, the combination treatment of NOB at a high dose (100 µM) and DTX (2.5 nM) further reduced cell proliferation by 32.1% and 36.5% compared to NOB and DTX respectively (Supplementary Fig. 6C). These results are consistent with those in MDA-MB-231 cells, suggesting a NOB function as a chemotherapy drug in TNBC.

### Synergistic antitumor effect of NOB in combination therapy

**Fig. 7:**
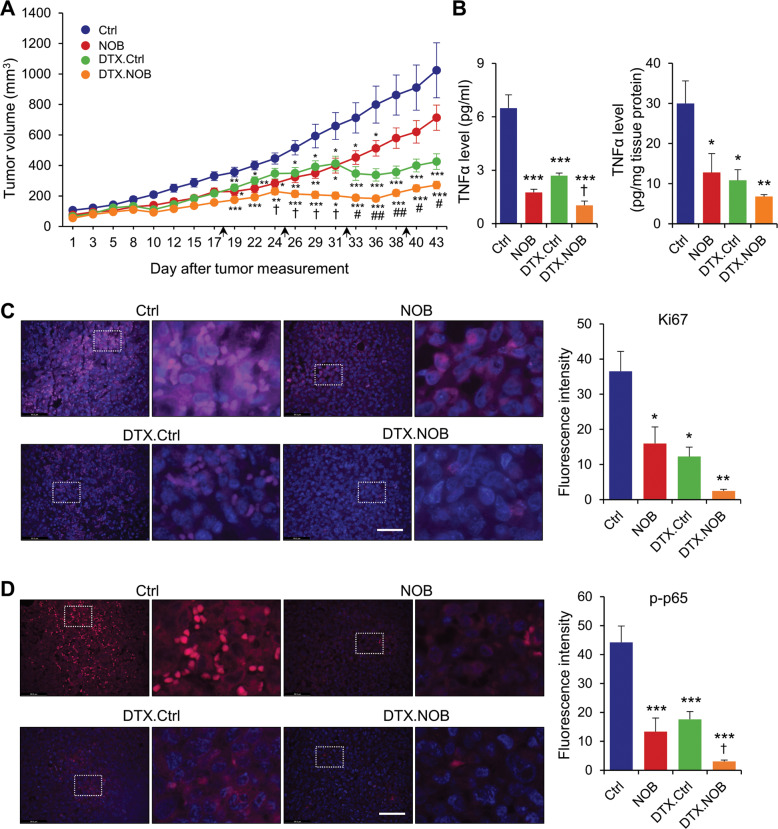
NOB suppresses tumorigenesis in MDA-MB-231 xenograft mice. **A** DTX and NOB showed synergistic inhibitory effects in MDA-MB-231 xenograft tumor growth (*n* = 6 for Ctrl and NOB, *n* = 7 for DTX.Ctrl, and *n* = 8 for DTX.NOB). The black arrows show DTX injection time point (18, 25, 32, 39 days). Data represent mean ± SEM. Two-way ANOVA with Tukey’s multiple comparisons test showed significant difference compared to Ctrl, *p < 0.05, **p < 0.01, and ***p < 0.001, compared with NOB, ^#^*p* < 0.05 and ^##^*p* < 0.01, and compared with DTX.Ctrl, ^†^*p* < 0.05, CI = 1.22. **B** TNF-α levels in plasma (left panel) and tumor (right panel) of MDA-MB-231 xenograft mice model. Data represent mean ± SEM. Two-way ANOVA with Tukey’s multiple comparisons test showed significant difference compared to Ctrl, *p < 0.05, **p < 0.01, and ***p < 0.001 and compared to DTX.Ctrl, ^†^*p* < 0.05. **C** Representative immunofluorescence images of the Ki67 (pink) and DAPI (blue) (×400 magnification, scale bar = 69.3 μm). Quantitative analysis of the percentage of Ki67 immunoreactive area. Data represent mean ± SEM. Two-way ANOVA with Tukey’s multiple comparisons test, *p < 0.05 and **p < 0.01. **D** Representative immunofluorescence images of the p-p65 (pink) and DAPI (blue) staining from representative images of the p-p65 (red) and DAPI (blue) immunofluorescence in the lesion area (×400 magnification, scale bar = 69.3 μm). Quantitative analysis of the percentage of p-p65 immunoreactive area. Data represent mean ± SEM. Two-way ANOVA with Tukey’s multiple comparisons test showed significant difference compared to Ctrl, ***p < 0.001 and compared to DTX.Ctrl, ^†^*p* < 0.05.

Finally, we tested the in vivo efficacy of the combination of DTX and NOB in mice bearing orthotopic MDA-MB-231 xenografts. Consistent with Fig. 1G, tumor growth was significantly retarded by NOB (Fig. 7A). DTX (indicated by arrow) alone significantly reduced tumor. Importantly, the DTX.NOB group showed the lowest tumor volume, with 73.5% reduction compared to the Ctrl and 36.2% compared to DTX alone (*p* < 0.05). In addition, the CI indicated the co-treatment of NOB and DTX shows synergistic effects on tumor growth inhibition (CI = 1.22; Supplementary Table 2). These findings suggested that the combination treatment of DTX and NOB might be a novel treatment strategy for TNBC patients.

TNF-α is highly increased in breast cancer, and its expression significantly correlated with the migration, invasion, and metastasis of breast cancer cells [65]. Since NF-κB activation induces TNF-α secretion, we measured TNF-α levels from plasma and tumor tissues. NOB and DTX reduced TNF-α in plasma and tumors compared to Ctrl. In particular, the TNF-α level was most significantly reduced by the DTX.NOB combination (*p* < 0.05) (Fig. 7B). Immunostaining of the proliferation marker Ki67 showed that, compared to the control, NOB alone reduced Ki67 level by 57.3% and the DTX.NOB combination by 93.3%, suggesting NOB markedly increases the efficacy of DTX (Fig. 7C). Consistent with cell line results (Fig. 4F, G), immunostaining showed strong reduction of p-p65 by NOB, and there is a combination effect in the DTX.NOB group (*p* < 0.05 compared to DTX.Ctrl) (Fig. 7D).

While TNF-α has been implicated breast cancer growth, it may also function in the development of antitumor immune response [66]. Therefore, we tested antitumor efficacy of NOB in a syngeneic xenograft model using DB7 cells in immune-competent mice. On day 29, the average tumor volume of the NOB group was reduced by 42.4% compared to Ctrl (Supplementary Fig. 7A). The TNF-α levels of plasma (65.8% vs Ctrl) and tumor (66.9% vs Ctrl) were significantly decreased by NOB (Supplementary Fig. 7B). NOB treatment significantly reduced Ki67 and p-p65 levels by 86.6% and 75.4%, respectively, relative to the control (Supplementary Fig. 7C, D). These results illustrate a strong anti-TNBC effect of NOB, alone or in combination, in cells and in vivo.

## Discussion

We demonstrate that ROR activation by NOB is effective at limiting TNBC cell growth in vitro as well as in xenografts, and NOB shows a synergistic effect with chemotherapeutic drugs, particularly DTX. Molecular studies reveal that NOB–ROR reduces tumor proliferation and inflammation responses via downregulating TNF-α activity and p65 nuclear localization in TNBC, and gain-of-function evidence demonstrates a requisite role of NOB repression of p65 against TNBC. Our study highlights a novel target and strategy for TNBC treatment and reveals the cellular mechanism underlying the anticancer effect of NOB.

While PMFs are generally well tolerated [6], their cellular mechanisms often are not well understood. We previously identified ROR as a direct, high-affinity target of NOB [18]. Building on this finding, we report here TNBC as a specific, sensitive target for the NOB–ROR axis, and identified IκBα/NF-κB as a key cellular pathway. We show that NOB was able to inhibit cell cycle progression and motility of TNBC cells in vitro, consistent with growth inhibitory effects of NOB against cancer cells [10, 15, 67]. Importantly, the observed effects were dependent on RORs, providing an important functional validation of the NOB–ROR axis [18, 19]. Consistent with our previous study [19], individual ROR knockdown was able to significantly inhibit NOB effects, perhaps due to reciprocal regulation between the RORs via RORE or other promoter elements [25, 42, 68]. Consistent with a previous study [59], we also demonstrate a direct transcriptional regulation of NOB–ROR on the IκB promoter and a role of NOB–ROR to suppress TNF-α induced p65 phosphorylation and nuclear localization (see below). These results together provide significant mechanistic insights for a NOB–ROR axis impinging on cancer and inflammation.

The role of NF-κB in tumorigenesis is well established [60, 61]. For example, the RelA/p65 NF-κB subunit is responsible for the transactivation of downstream genes involved in the modulations of cell cycle distribution, cell survival, and apoptosis [69]. In accordance, p65 activation is an important sign of resistance to neoadjuvant chemotherapy in breast cancer patients [70]. Particularly for TNBC, p65 overexpression induced NF-κB transcriptional activity and inhibited apoptosis triggered by celecoxib, a selective cyclooxygenase (COX)-2 inhibitor, in MDA-MB-231 cells [71]. Here, overexpression of p65 abolished the NOB effect on MDA-MB-231 cell proliferation and motility, providing important evidence that repression of NF-κB signaling is required for the anticancer effect of NOB. TNF-α upregulation of the NF-κB pathway is a major regulatory step for inflammation, including in the tumor microenvironment [72]. Here, we found that NOB dampened TNF-α-induced p65 phosphorylation and NF-κB nuclear localization in MDA-MB-231 cells and xenograft tumors. Previously, RORα overexpression abrogated glioma tumorigenesis through reducing TNF-α-mediated NF-κB signaling [73]. Consistently, we show that ROR overexpression inhibited proliferation and motility in MDA-MB-231 cells, which was further enhanced by NOB.

TNBC is heterogeneous and only a subset of patients responds favorably to standard chemotherapy [1, 4]. While combination of chemo- and radio-therapy is the current standard treatment options for TNBC, there is an urgent need for new targets and therapeutic strategies including combination therapies. DTX has been employed in a combination therapy with JMR-231, a growth hormone-releasing hormone (GHRH) antagonist, which reduced tumor growth by 71.6% in MDA-MB-231 xenograft mice [74]. Here, we revealed an ROR-dependent mechanism suppressing NF-κB signaling for a specific anti-TNBC effect of NOB, a promising chemopreventive agent. Importantly, in vitro and xenograft studies revealed superior antitumor efficacies by the DTX.NOB combination compared to NOB or DTX alone, which correlated with a similar effect to attenuate TNF-α levels in plasma and tumors. Our study is consistent with a previous study where NOB enhanced efficacies of several chemotherapeutic drugs including DTX against cancer cells that have acquired multidrug resistance [16]. Together, our results highlight a potential role of NOB as an anticancer agent, by itself or in combination.

A number of studies have illustrated functional interactions between NF-κB signaling and circadian clocks [75–78]. However, while our initial studies identified NOB as a circadian clock-enhancing compound targeting RORs [18, 79], MDA-MB-231 cells did not display sustained circadian rhythms, consistent with other results [53]. Previously, we showed that preventive efficacies of NOB against metabolic disorders requires a functional circadian clock [18]. Whereas clock gene expression has been detected in TNBC, its expression levels tend to be dysregulated, including *RORs* [51, 53, 80]. NOB appeared not to have rejuvenated circadian rhythms in MDA-MB-231 cells, suggesting that the NOB effect is mediated by RORs in a clock-independent manner [26, 27]. However, it remains to be investigated whether ROR activation by NOB intersects with cellular pathways that may require individual clock components present in arrhythmic TNBC cells. In that regard, it is interesting to note that NOB inhibition of xenografts is greater in immune-competent host mice than in nude mice (Supplementary Fig. 7A vs Fig. 7A). Future studies should examine whether the systemic NOB effects on the host, including circadian rhythms and the immune system, may synergize with the local effects on the tumor to achieve optimal outcomes.

In summary, we delineate an ROR–IκBα/NF-κB mechanistic pathway that underlies the anti-cancer effects of NOB, alone or in combination, against TNBC. Our data suggest a possible crosstalk between anti-inflammatory and anti-cancer function of this versatile PMF. These results highlight NOB–ROR as a novel therapeutic strategy against TNBC and inflammation.

## Supplementary information

Supplemental Material

Original Western blots

Reproducibility checklist

## Data Availability

All raw data or information related to the current work are available from the corresponding authors upon reasonable request.
