# Supplementary material for: ROR activation by Nobiletin enhances antitumor efficacy via suppression of IκB/NF-κB signaling in triple-negative breast cancer
Source: Cell Death Dis. 2022 Apr 19;13(4):374. doi: 10.1038/s41419-022-04826-5 (PMC9018867; doi:10.1038/s41419-022-04826-5)
Supplement: Supplementary file 1 — Supplemental Material [file 41419_2022_4826_MOESM1_ESM.pdf]

Supplementary Information

ROR activation by Nobiletin enhances anti-tumor efficacy via suppression of IκB/NF-κB signaling in triple-negative breast cancer

Eunju Kim, Yoon-Jin Kim, Zhiwei Ji, Jin Muk Kang, Marvin Wirianto, Keshav Raj Paudel, Joshua A. Smith, Kaori Ono, Jin-Ah Kim, Kristin Eckel-Mahan, Xiaobo Zhou, Hyun Kyoung Lee, Ji Young Yoo, Seung-Hee Yoo, Zheng Chen

This SI file contains 7 supplementary figures/legends and 2 supplementary tables.

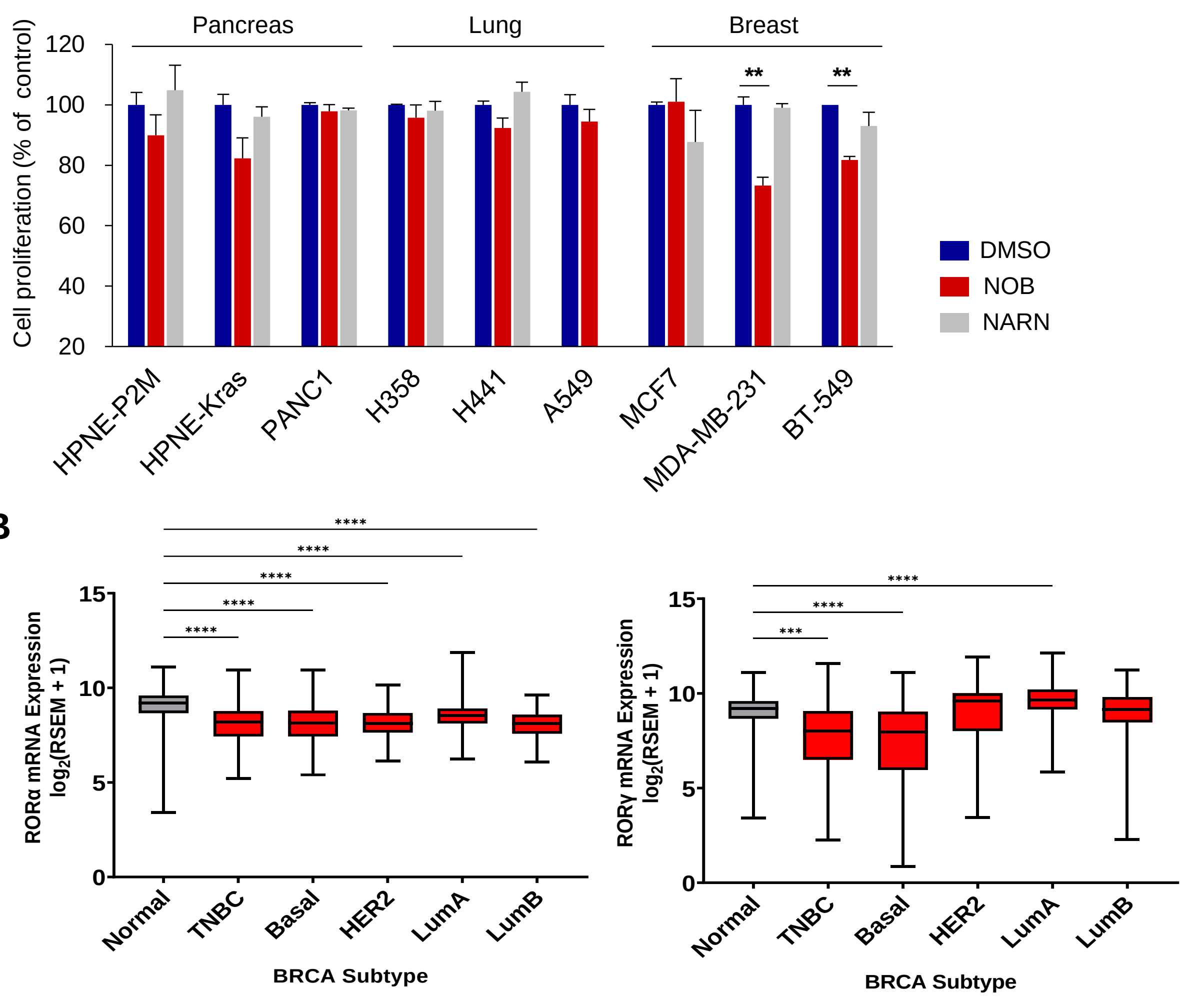

C

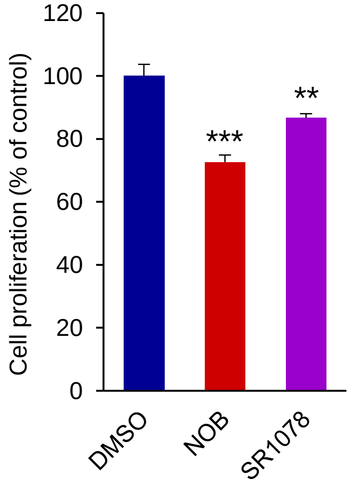

Supplementary Fig. 1. Anti-TNBC effects of NOB-ROR. A. Screening of NOB inhibitory effects in cancer cell lines. Pancreas, lung and breast cancer cell lines were seeded in 96-well plate overnight and treated with 10 μM NOB. Cell proliferation was measured by WST-1 assays 72 hrs after NOB treatment. Data represent mean ± SEM. Two-tailed Student's t-test, ** $p<0.01$ vs. control. **B.** TCGA database search revealed that *RORA* and *RORC* expression was significantly reduced in TNBC and some other subtypes. **C.** RORα/γ agonists decreased cell proliferation. MDA-MB-231 cells were treated with NOB 10 μM and SR1078 10 μM for 72 hrs. Cell proliferation was determined by WST-1 assays. DMSO (0.1%) was used as a solvent control. Data represent mean ± SEM. One-way ANOVA with Tukey's post hoc test. Significantly different compared with DMSO ** $p<0.01$ and *** $p<0.001$.

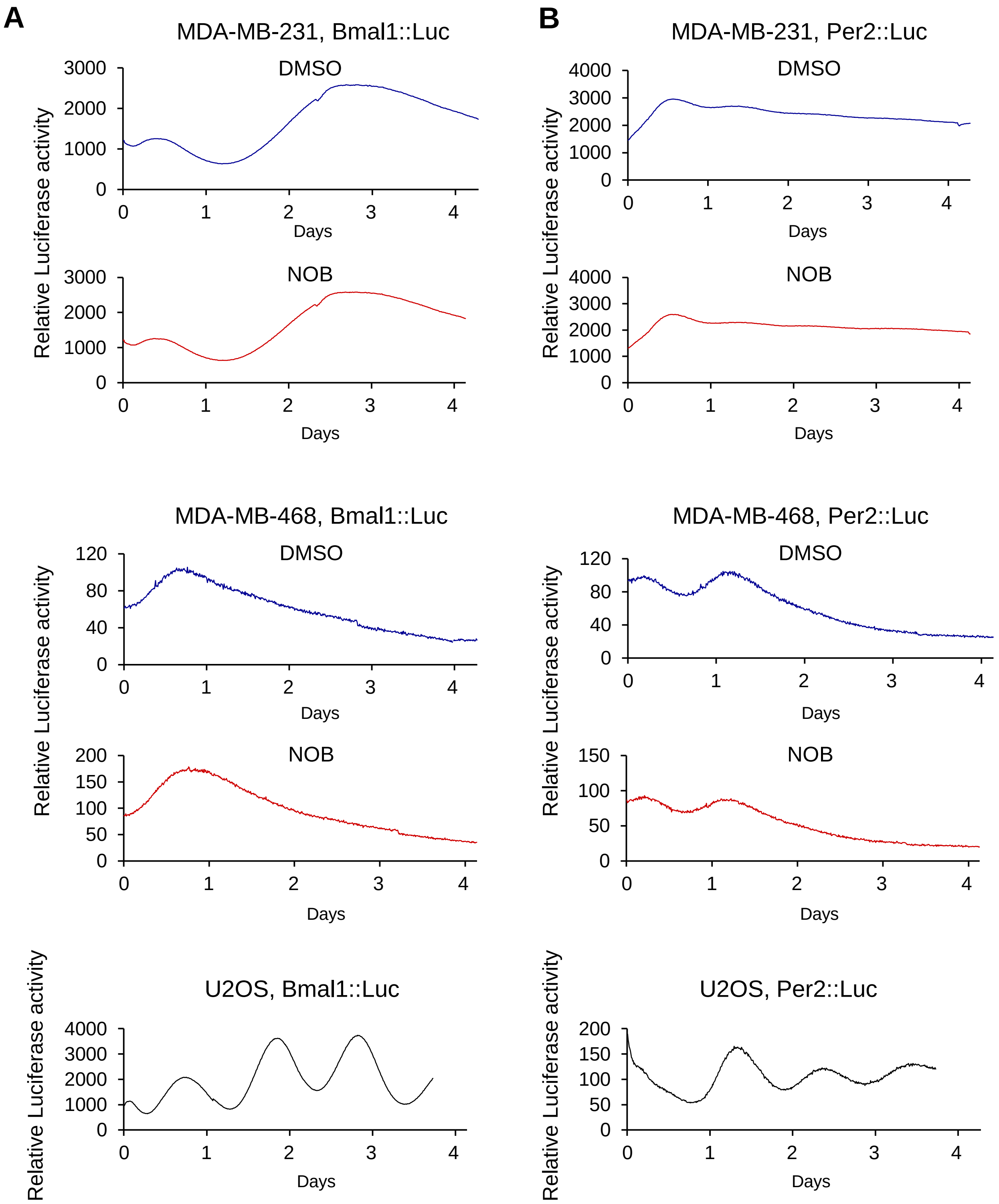

Supplementary Fig. 2. Absence of robust circadian rhythms in MDA-MB-231 and MDA-MB-468. A-B. MDA-MB-231 or MDA-MB-468 cells were transfected with Bmal1::Luc (**A**) and Per2::Luc (**B**) reporter constructs and stable cell lines were established. Cells were synchronized and treated with NOB 10 µM. Real-time bioluminescence recordings were analyzed by Lumicycle analysis program (Actimetrics). Lower panels: U2OS cells transfected with these reporter constructs show circadian rhythms.

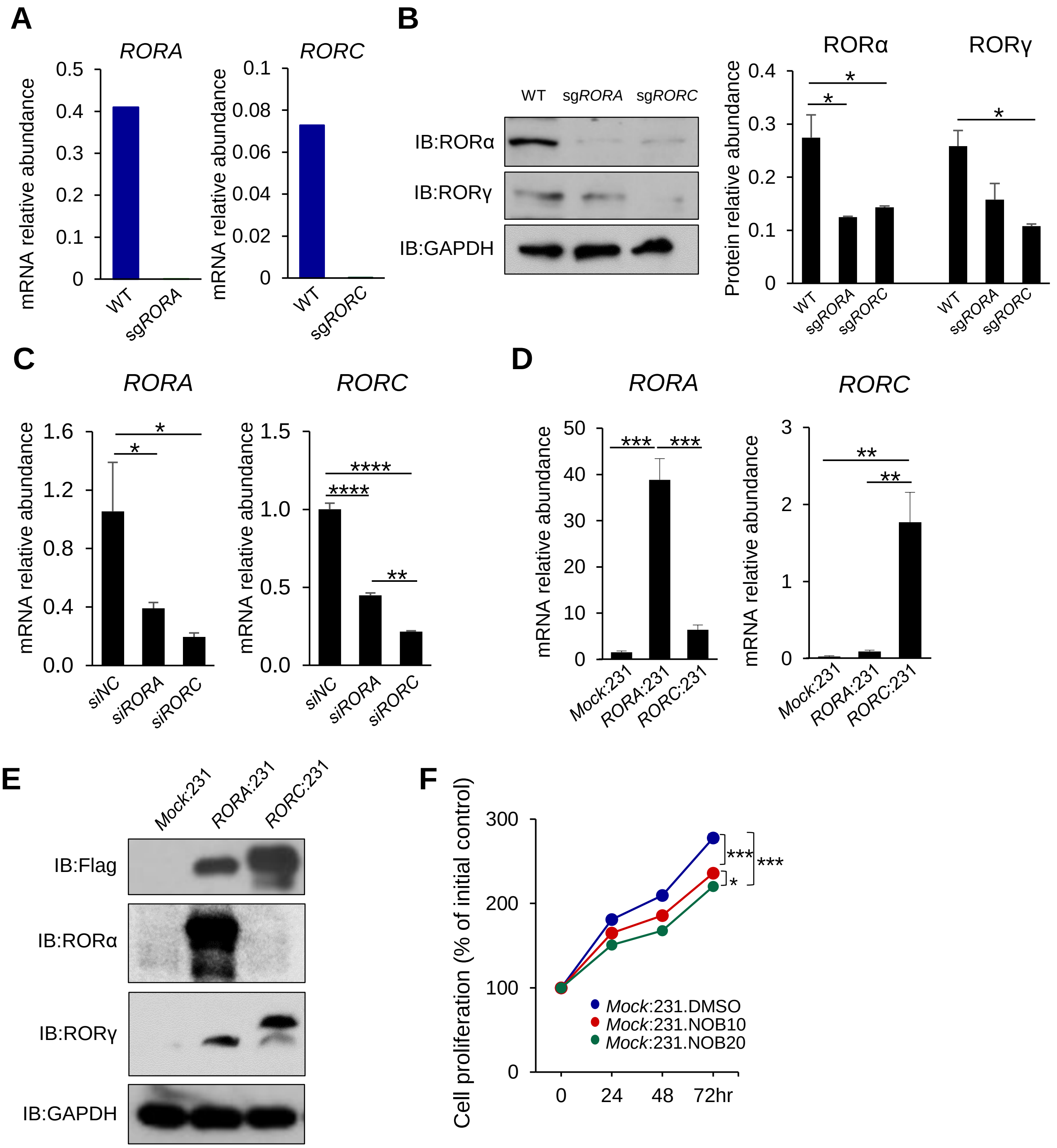

A
RORA
RORC
mRNA relative abundance
WT
sgRORA
sgRORC
B
WT sgRORA sgRORC
IB:RORα
IB:RORγ
IB:GAPDH
RORα
RORγ
Protein relative abundance
C
RORA
RORC
siNC
siRORA
siRORC
D
Mock:231
RORA:231
RORC:231
E
IB:Flag
F
Cell proliferation (% of initial control)
0 24 48 72hr
Mock:231.DMSO
Mock:231.NOB10
Mock:231.NOB20

Supplementary Fig. 3. Loss- and gain-of-function studies of RORs. A. Validation of *RORA* and *RORC* expression in CRSPR knockdown cells by qPCR analysis. *GAPDH* was used as a reference gene. **B.** Validation of *RORA* and *RORC* expressions in knockdown cells by Western blotting using RORα and RORγ antibodies. One-way ANOVA with Tukey's multiple comparisons test, * p<0.05 and *** p<0.001. **C**. Validation of *RORA* and *RORC* expressions in si*RORA* and si*RORC* MDA-MB-231 cells by qPCR. One-way ANOVA with Tukey's multiple comparisons test, * p<0.05, ** p<0.01, and **** p<0.0001. **D**. Validation of *RORA* and *RORC* expressions in *RORA* or *RORC* over-expression MDA-MB-231 cells by qPCR. One-way ANOVA with Tukey's multiple comparisons test, * p<0.05, ** p<0.01, and *** p<0.001. **E.** RORα or RORγ protein expression in *RORA* or *RORC* over-expression MDA-MB-231 cell lines. Western blotting was performed using Flag, RORα, and RORγ antibodies. **F.** NOB inhibited cell proliferation of MDA-MB-231 cells transfected with the empty vector (P3Xflag-CMV10-Neo^{r}) dose-dependently. Mock indicates P3Xflag-CMV10-Neo^{r} (Empty vector) as the control. DMSO was used as a solvent control, NOB10, 10 µM; NOB 20, 20 µM. Data represent mean ± SEM. One-way ANOVA with Tukey's multiple comparisons test, * p<0.05 and *** p<0.001.

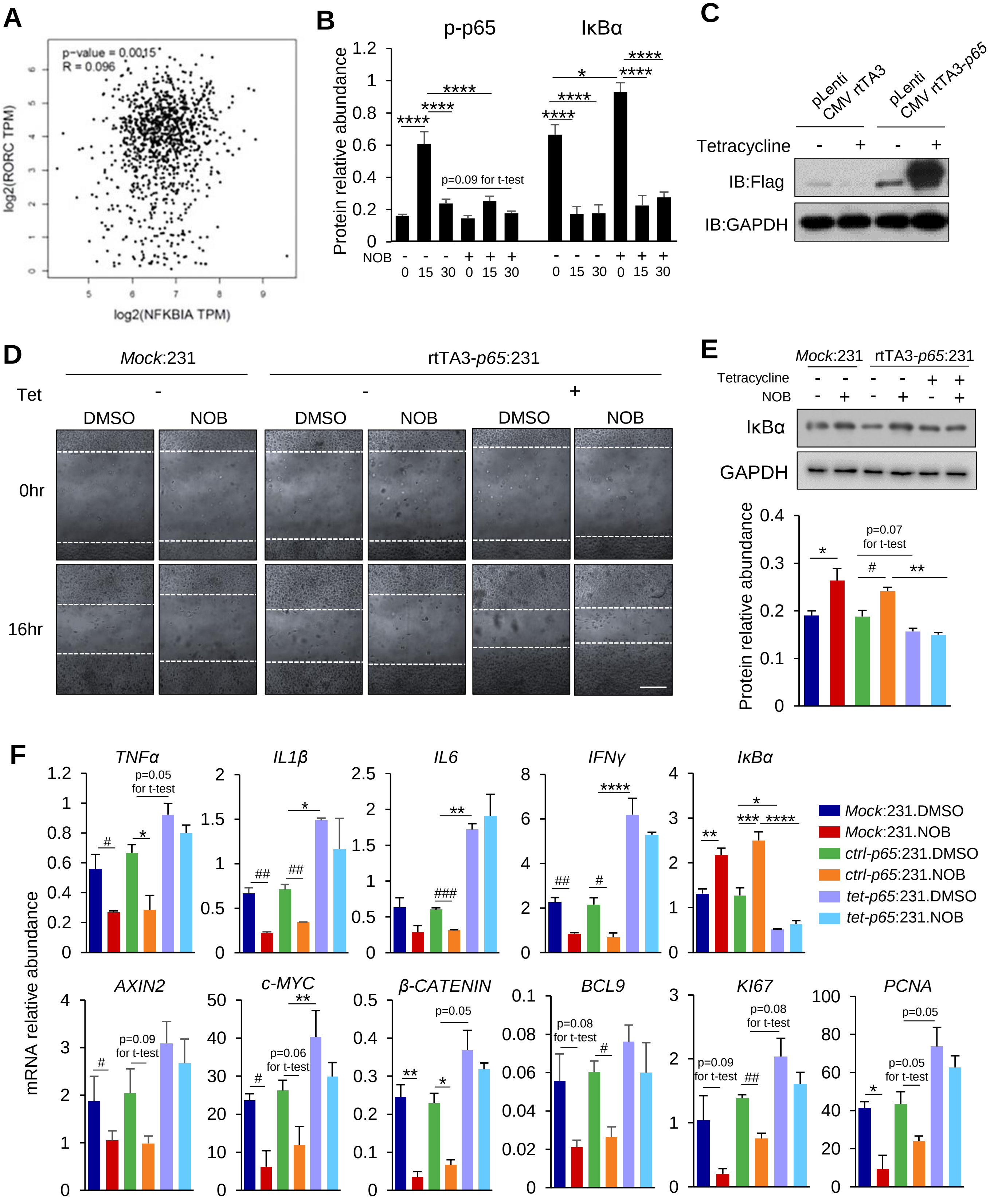
A
p-value = 0.0015
R = 0.096
log2(RORC TPM)
log2(NFKBIA TPM)
B
p-p65
IκBα
Protein relative abundance
p=0.09 for t-test
NOB
C
pLenti CMV rtTA3
pLenti CMV rtTA3-p65
Tetracycline
IB:Flag
IB:GAPDH
D
Mock:231
rtTA3-p65:231
Tet
DMSO
NOB
0hr
16hr
E
Mock:231
rtTA3-p65:231
Tetracycline
NOB
IκBα
GAPDH
Protein relative abundance
p=0.07 for t-test
F
TNFα
IL1β
IL6
IFNγ
IκBα
AXIN2
c-MYC
β-CATENIN
BCL9
KI67
PCNA
mRNA relative abundance
p=0.05 for t-test
p=0.09 for t-test
p=0.06 for t-test
p=0.05
p=0.08 for t-test
p=0.09 for t-test
p=0.08 for t-test
Mock:231.DMSO
Mock:231.NOB
ctrl-p65:231.DMSO
ctrl-p65:231.NOB
tet-p65:231.DMSO
tet-p65:231.NOB

Supplementary Fig. 4. Regulation of the NF-κB pathway by NOB. A. TCGA database analysis revealed a significant correlation of *RORC* and *NFKBIA* (encoding IκBα) expression in breast cancer. **B**. Quantification of p-p65 and IκBα expressions in Fig. 4D. Data represent mean ± SEM. Two-way ANOVA with Sidak's multiple comparisons test, * $p<0.05$; **** $p<0.0001$. **C**. Inducible p65 expression using pLenti CMV rtTA3-*p65* in MDA-MB-231 cell line. **D**. Representative images of cell motility measured by wound healing assays (x 100 magnification, scale bar = 277.3 μm). DMSO was used as a vehicle control; NOB, 20 μM. **E**. IkBα expression in MDA-MB-231 cells after p65 expression induction. **F**. mRNA expression of cancer related-genes for pro-inflammatory cytokines, Wnt/β-catenin signaling, and cell proliferation. Data represent mean ± SEM. Two-way ANOVA with Tukey's multiple comparisons test showed significant difference, * $p<0.05$; ** $p<0.01$; **** $p<0.0001$. Student t-test. Significantly different, # $p<0.05$; ## $p<0.01$; ### $p<0.001$.

A

	Gene name
Positive >2-fold change	TTC25, SLC25A41, TLE6, ICAM2, SLC25A21-AS1, LINC01173, ZNF132, PADI3, TNIP3, FPR1, SLC38A5, CCR7, C3AR1, HPD, CFAP58, MKRN3, LACTB2-AS1, QRFP, TUBAL3, CFAP69, BDKRB1, MIR5689HG, C9orf43, LLPH-DT, CPA5, GAL3ST4, ACSM4, CA3, MIR181A2HG, GRIP1, CCDC180, C6orf99, HHIPL2, CXCL3, FLNB-AS1, IL3RA, LINC02601, SH2D1B, MIR4292, USP50, USP27X-AS1, FAM166B, CCDC173, POPDC2, SERPIND1, C4orf47, STK4-AS1, ACRV1, LINC01812, SIRT4, S100A6, CLEC18B, LINC01179, FOXO6-AS1, ABI3BP
Negative >2-fold change	ABCA12, MYO16-AS1, ATP13A5, RGMB-AS1, NLRP14, ADAM21, LINC01714, MS4A4A, LRRC19, TTLL13P, STAG3, KLHL10, XKRX, BTBD18, DDIT4L, ABCA4, TSTD2, STRA6, C3orf49, PP2D1, REL, ERCC5, FARSA-AS1, LYG1, MLANA

B

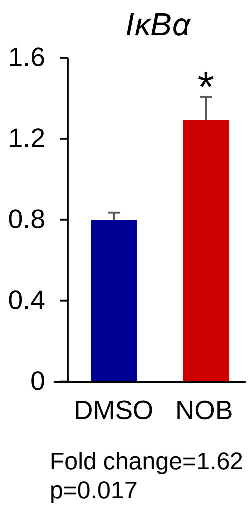

Supplementary Fig. 5. RNA-seq analysis using NOB-treated MDA-MB-231 cells.
A. Overlapping genes between differentially expressed genes (DEGs) and RORγ target genes (GSE126380) in the GEO database in breast cancer. DEGs showing 2-fold or more changes in expression are shown. **B.** qPCR validation of IκBα mRNA induction by NOB in MDA-MB-231 cells.

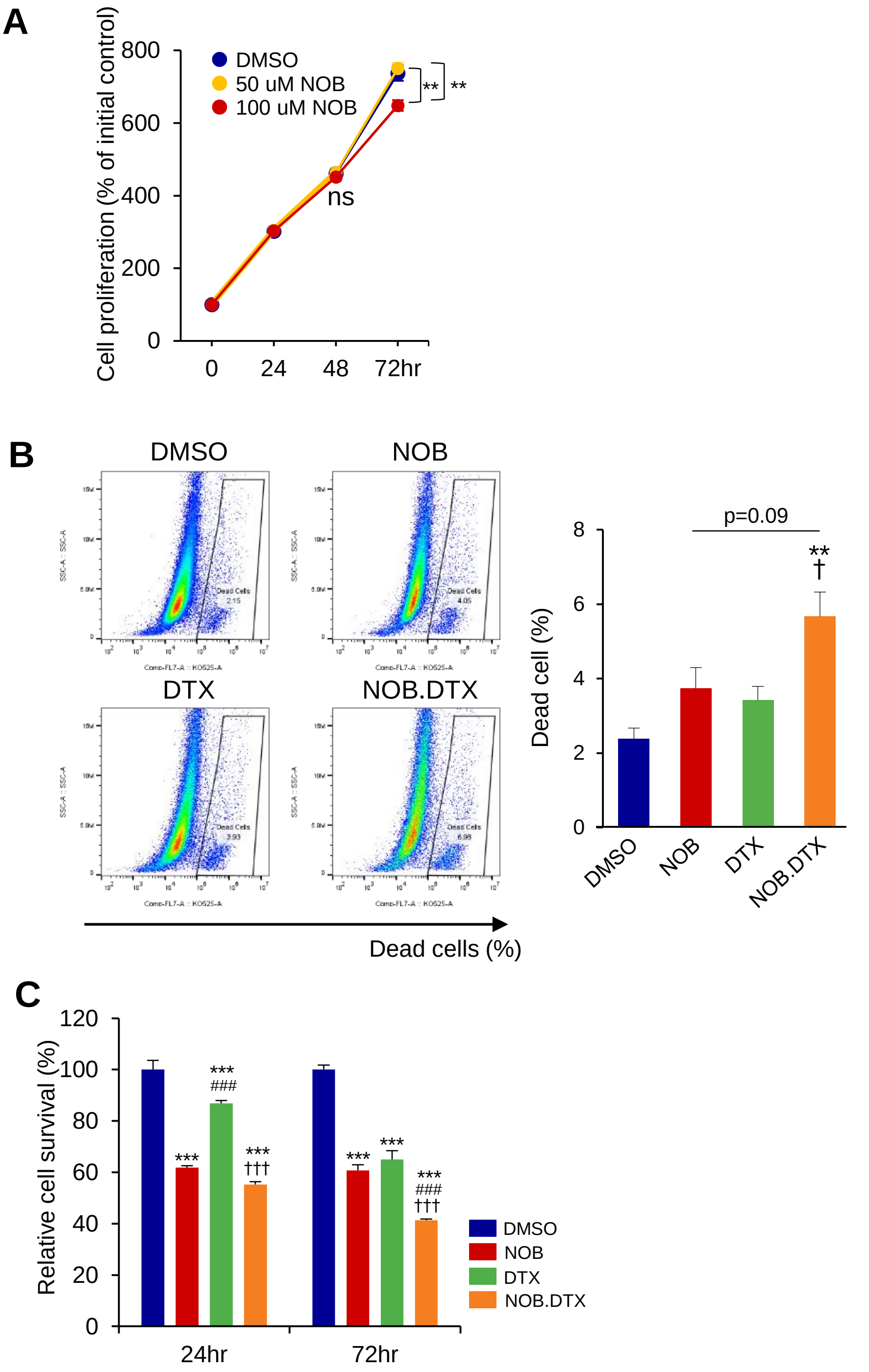

Supplementary Fig. 6. Combination studies show the efficacy of NOB with DTX in MDA-MB-468 and DB7 cells. A. Cell proliferation of the non-tumor MCF10A cells was measured by WST-1 assays for 72 hrs after NOB (50 or 100 µM) treatment. One-way ANOVA with Tukey's post hoc test. ** $p<0.01$. **B.** MDA-MB-468 cells were treated with the NOB (50 µM) and DTX (2.5 nM) combination for 48 hrs and cell death was determined by FACS analysis. Left panel; Representative images; right panel; quantification. Data represent mean ± SEM. Two-way ANOVA with Tukey's multiple comparisons test showed significant difference compared to DMSO, ** $p<0.05$, DTX, † $p<0.05$. **C.** DB7 cells were treated with the NOB (100 µM) and DTX (2.5 nM) combination for 24 and 72 hrs and cell proliferation was determined by MTT assay. Data represent mean ± SEM. Two-way ANOVA with Tukey's multiple comparisons test showed significant difference compared to DMSO, *** $p<0.001$, compared to NOB, ### $p<0.001$, and compared to DTX.NOB, ††† $p<0.001$.

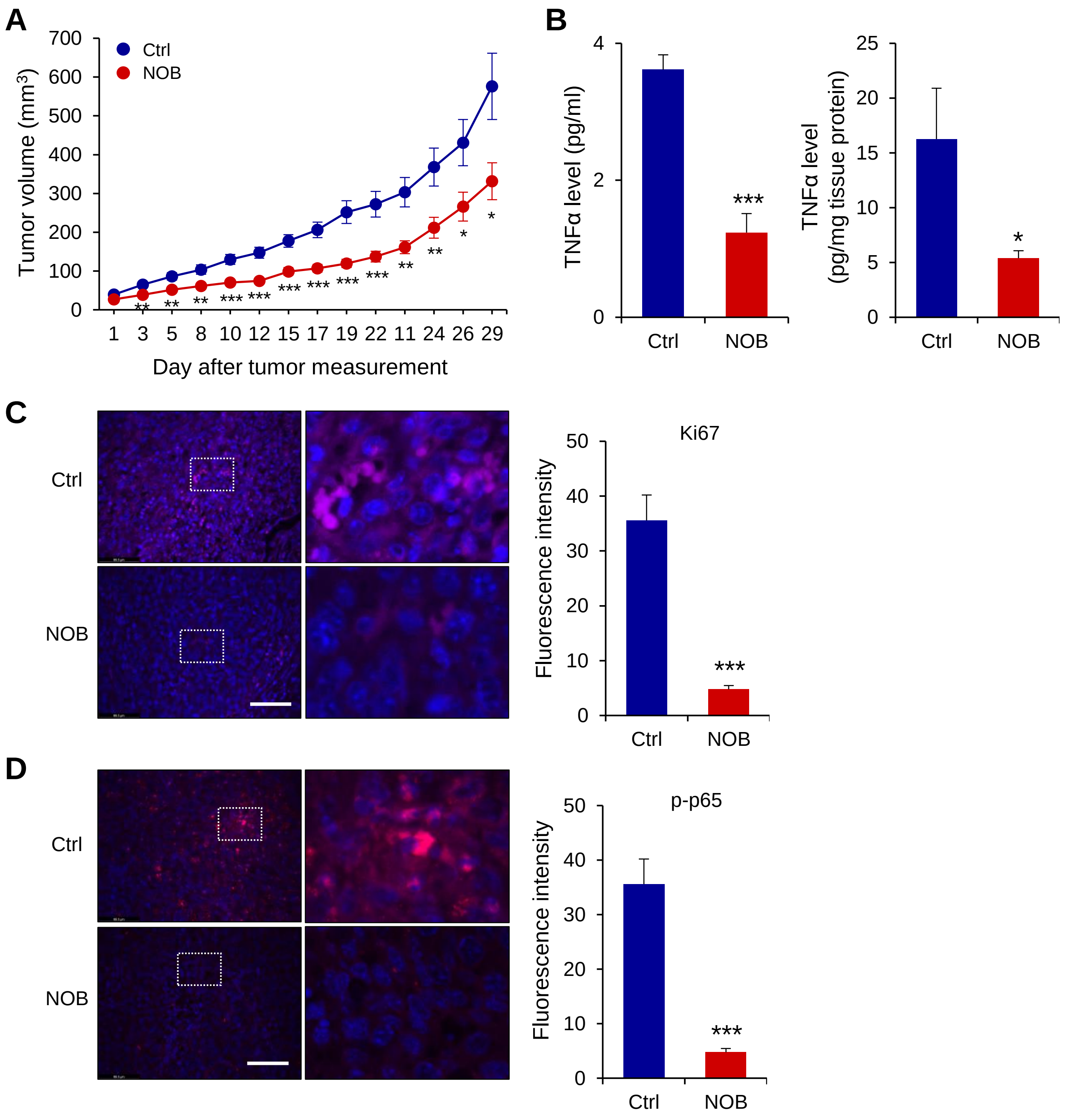

Supplementary Fig. 7. NOB suppresses tumorigenesis in DB7 xenograft mice. A. NOB induced anti-tumorigenesis effects in DB7 xenograft mice (n=17 for Ctrl and n=18 for NOB). Data represent mean ± SEM. Student *t*-test, * $p<0.05$, ** $p<0.01$, and *** $p<0.001$. **B.** TNF-α level of plasma (left panel) and tumor (right panel) of DB7 xenograft mice model. We randomly chose eight samples per group for ELISA measurement. Data represent mean ± SEM. Student *t*-test, * $p<0.05$ and *** $p<0.001$. **C.** NOB reduced Ki67 expression from DB7 xenograft tumor. Representative images of the Ki67 (pink) and DAPI (blue) immunofluorescence in the lesion area (x 400 magnification, scale bar = 69.3 μm). Quantitative analysis of the percentage of Ki67 immunoreactive area. Data represent mean ± SEM. Student *t*-test. Significantly different compared with Ctrl, *** $p<0.001$. **D.** NOB reduced p-p65 expression from DB7 xenograft tumor. Representative images of the p-p65 (red) and DAPI (blue) immunofluorescence in the lesion area. (x 400 magnification, scale bar = 69.3 μm). Quantitative analysis of the percentage of p-p65 immunoreactive area. Data represent mean ± SEM. Student *t*-test. Significantly different compared with Ctrl, *** $p<0.001$.

Supplementary table 1. Primer sequences for qPCR analysis.

	Forward	Reverse
RORA	CTTCTTTCCCTACTGTTCGTTC	GCTCTTCTCTCAAGTATTGGC
RORC	GTGGGGACAAGTCGTCTGG	AGTGCTGGCATCGGTTTCG
TNFα	CTCTTCTGCCTGCTGCACTTTG	ATGGGCTACAGGCTTGTCACTC
IL1β	CACAGACCTTCCAGGAGAATG	GCAGTTCAGTGATCGTACAGG
IL6	AGACAGCCACTCACCTCTTCAG	TTCTGCCAGTGCCTCTTTGCTG
IFNγ	GAGTGTGGAGACCATCAAGGA	GCTTTGCGTTGGACATTCAAGTC
Axin2	TACACTCCTTATTGGGCGATCA	AAGTTCGGAACAGGTAAGCAC
c-MYC	GGAACGAGCTAAAACGGAG	GGCCTTTTCATTGTTTTCCAACT
β-CATENIN	CACAAGCAGAGTGCTGAAGGTG	GATTCCTGAGAGTCCAAAGACAG
BCL9	TCCAGCTCGTTCTCCCAACTTG	GATTGGAGTGAGAAAGTGGCTGG
KI67	CAAGGAACAGCCTCAACCAT	ACCAAGCTTTGTGCCTTCAC
PCNA	CAAGTAATGTCGATAAAGAGGAGGA	TTCAGGTACCTCAGTGCAAAAG
GAPDH	CGACCACTTTGTCAAGCTCA	AGGGGTCTACATGGCAACTG

Supplementary Table. 2. Mean tumor growth inhibition rate in breast cancer mice on Day 45 since the 1st measurement.

	MGI [a]	Expected [b]	CI [c]
Ctrl			
NOB	0.69		
DTX.Ctrl	0.36		
DTX.NOB	0.21	0.26	**1.22**

[a] Mean tumor growth inhibition rate (MGI) = tumor volume on day 43 - tumor volume on day 0 of the treated group/tumor volume on day 43 - tumor volume on day 0 of the control group

[b] Expected growth inhibition rate = growth inhibition rate of NOB x growth inhibition rate of DTX.Ctrl.

[c] Combination index (CI) = expected growth inhibition rate/observed growth inhibition rate (MGI).

[d] An index >1.1 indicates a synergistic effect, between 0.9 and 1.1 indicates an additive effect, and <0.9 indicates a less than additive effect.
