# Supplementary figures and images for: ROR activation by Nobiletin enhances antitumor efficacy via suppression of IκB/NF-κB signaling in triple-negative breast cancer
Source: Cell Death Dis. 2022 Apr 19;13(4):374. doi: 10.1038/s41419-022-04826-5 (PMC9018867; doi:10.1038/s41419-022-04826-5)

Figure 2

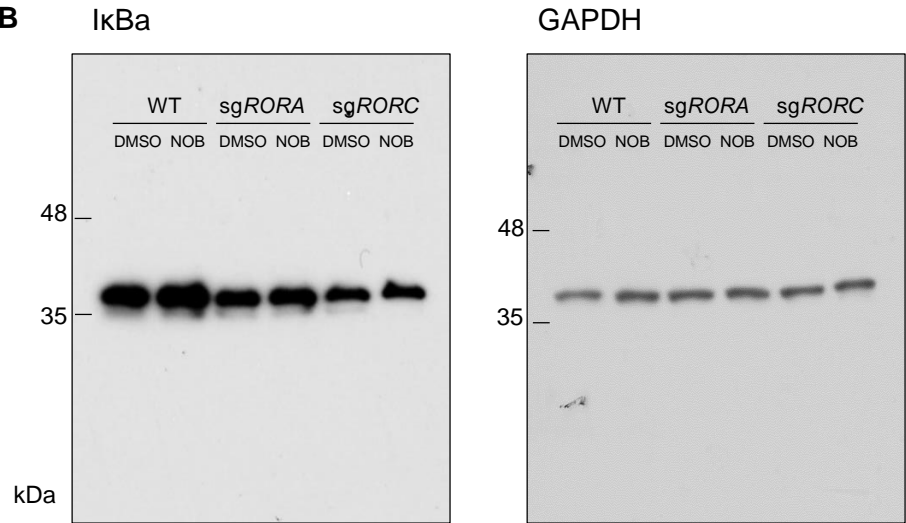



Figure S3

**B**

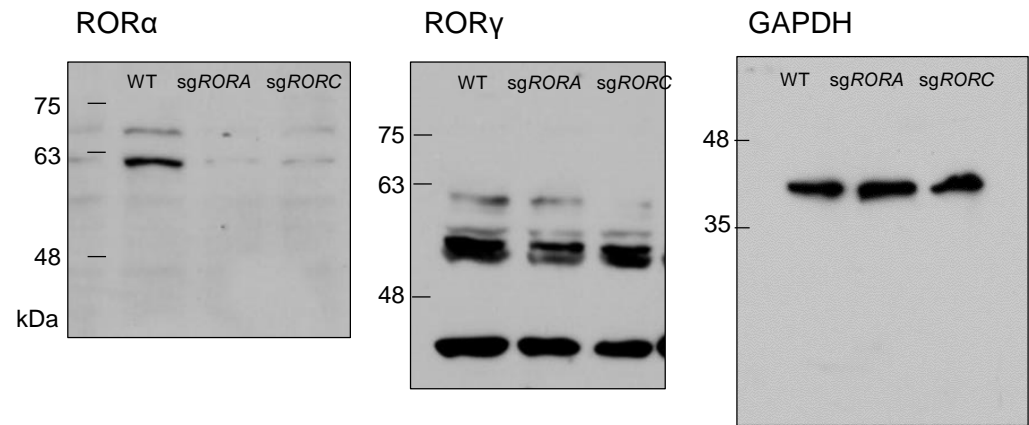

**E**

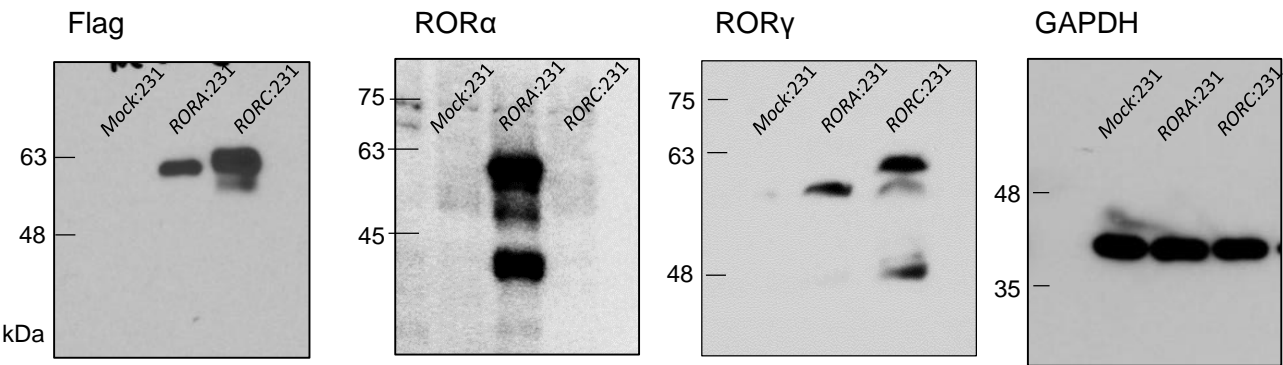

Figure S4

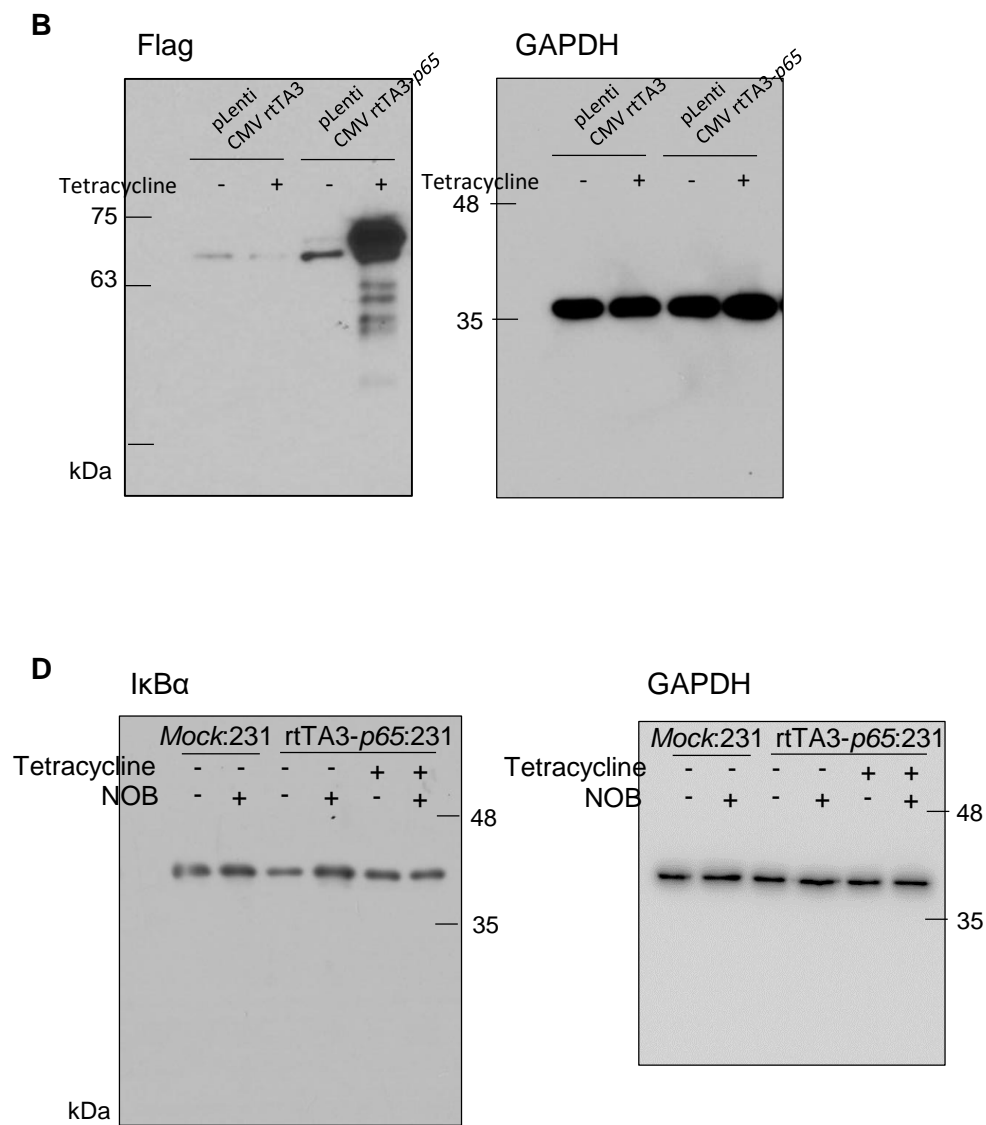

Supplement: Supplementary file 2 — Original Western blots [file 41419_2022_4826_MOESM2_ESM.pdf]
